# Optimization of Mucoadhesive Oral Films Containing Olive Leaf Extract and Microencapsulated Thyme Essential Oil With Potential Antimicrobial Activity

**DOI:** 10.1002/fsn3.4603

**Published:** 2025-01-31

**Authors:** Kubra Goktas, Dilek Yalcin, Cansu Erdem, Beyza Tutku Bicakci, Oguz Bayraktar

**Affiliations:** ^1^ Department of Bioengineering Ege University Izmir Turkey

## Abstract

In this study, developing mucoadhesive oral films with optimized olive leaf extract (OLE) and microencapsulated thyme essential oil (TEO) contents was aimed to create a novel oral delivery system with desirable film properties as well as enhanced bioactivities. For this, TEO‐containing sodium alginate/okra gum microcapsules were first fabricated at optimized electrospray conditions, where optimization was performed for three parameters, which are the flow rate of the emulsion solution, the potential difference, and the distance between the nozzle of the sample container and the collector plate, through three‐level centered Box–Behnken design. Prior to the inclusion of the OLE in custom‐designed mucoadhesive films, polymeric components of the films were also optimized through a separate multi‐variable, three‐level Box–Behnken experimental design by which the effect of mixing ratios of Carbopol 934, hydroxypropyl methylcellulose and polyethylene glycol on viscosity, solution pH, film pH, disintegration time, thickness, and weight were elaboratively investigated. The resulting films, containing OLE and microencapsulated TEO, obtained through solvent casting were characterized for pH, viscosity, mass uniformity and thickness, dispersion, tensile strength, folding endurance, contact angle, in vitro mucoadhesion time, and ex vivo mucoadhesion force. Results showed that the film had good tensile strength, swelling capacity, compatibility with oral mucosa, and flexibility. It also demonstrated desirable stability and adhesion ability. Regarding bioactivity, the film was found to have desirable antioxidant capacity and it was non‐toxic to fibroblast cells when diluted to certain concentration. Moreover, antiviral studies indicated reductions of 32.39% and 54.29% against *Poliovirus Type 1* and *Murine Norovirus*, respectively, while preliminary antibacterial tests showed no significant reduction in certain bacteria within 1 min, but a logarithmic reduction in *Staphylococcus aureus* was observed. Despite favorable mucoadhesion properties and physical attributes, further investigation is needed to enhance the antimicrobial effectiveness of the films for their potential utilization in drug delivery systems.

## Graphical abstract

The final optimized mucoadhesive film had good tensile strength, swelling capacity, compatibility with oral mucosa, and flexibility. It also demonstrated desirable stability and adhesion ability. Regarding bioactivity, the film was found to have desirable antioxidant capacity, and it was non‐toxic to fibroblast cells when diluted to certain concentration. Moreover, antiviral studies indicated reductions of 32.39% and 54.29% against *Poliovirus Type 1* and *Murine Norovirus*, respectively, and a logarithmic reduction in *Staphylococcus aureus* was observed.

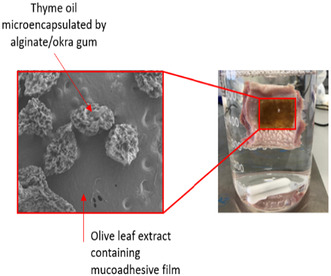

## 1 Introduction

Any abnormal change in the surface of the oral mucosa, manifesting as pigmented and ulcerative features, any form of swelling or developmental defects have been defined as oral mucosal lesions (OMLs). Since 2003, OML has been recognized as a major public health issue globally by the World Health Organization (WHO) as the condition of the oral mucosa is often indicative of a patient's overall health (Bahare Salehi et al. 2019). There are several factors that can cause an OML, including pathogenic infections, thermal or physical trauma, immune system deficiencies, systemic diseases, aging, and chronic behaviors such as alcohol and tobacco use. Among them, the cytotoxic effects of cancer chemotherapeutics are suspected to be one of the well‐known factors, as 40% to 70% of cancer patients develop certain OMLs (Ikeuchi‐Takahashi, Kobayashi, and Onishia 2017). Although the pathogenesis of OMLs such as mucositis is quite complex and still unrevealed, it is believed that reactive oxygen species generated by cancer chemotherapeutics initiates oxidative stress, which in turn leads to an OML (Showraki et al. 2016).

There are a number of agents, currently used for managing chemotherapy related‐OML, such as steroids, vitamins, antimicrobial agents, and particularly synthetic antioxidants owing to their radical scavenging potential (Ikeuchi‐Takahashi, Kobayashi, and Onishia 2017; Zhang et al. 2019). However, due to the adverse side effects as well as low bioefficacy of these therapeutics, developing new treatments with an innovative and effective delivery strategies are still highly warranted. In the treatment of the oral cavity diseases, one particular problem, which is common to many drug delivery systems, is the short residence time at the site of application. To overcome this problem, using bioadhesive polymers as delivery vehicles is gaining a remarkable attention in recent years, since an effective buccal release system should be flexible and bioadhesive by nature ensuring drug/therapeutics remains in the oral cavity for a desirable period (Asati, Jain, and Choubey 2019; Alawdi and Solanki 2021; Fonseca‐Santos and Chorilli 2018).

To date, bioadhesive mucosal therapeutics have been developed in the form of tablets, gels, patches, and polymeric films. The desirable characteristics of a mucoadhesive dosage form include high drug‐loading capacity, sustained drug release, increased residence time of the drug, protection of delicate structures such as protein‐based drugs, improved bioavailability, smooth surface, and ease of application (Asati, Jain, and Choubey 2019; Punitha and Girish 2010; Wong, Yuen, and Peh 1999; Ways, Lau, and Khutoryanskiy 2018). With this respect, buccal films are mostly preferable over the other dosage forms. Although mucoadhesive systems provide diverse benefits, most formulations still require optimizations in terms of both film‐making properties and increased bioactivities.

Many synthetic and biopolymers exhibit mucoadhesive properties, thereby playing a significant role in the sustained release of drugs from different mucous membranes such as ocular, nasal, buccal, or vaginal (Cazorla‐Luna et al. 2021). Polyvinyl alcohol (PVA), polyacrylic acids (i.e., Carbopol) and polyethylene glycols (PEGs) are common biocompatible synthetic polymers while alginates, alkylcelluloses, and chitosan are common natural biopolymers used in the formulations of mucoadhesive delivery systems due to their excellent film making and stabilizing properties as well as controllable hydrophilicity, which is a key parameter for modulating drug release (Ikeuchi‐Takahashi, Kobayashi, and Onishia 2017; Köse, Gümüş Işik, and Bayraktar 2021).

Among them, alginate is a natural, non‐branched polyanionic polysaccharide. Due to its biocompatible nature, pH responsiveness, biodegradability, non‐irritant properties, and non‐toxicity, alginate finds extensive applications in drug delivery and biomedical field from wound dressings to tissue regeneration (Shtenberg et al. 2018; Sosnik 2014; Chia et al. 2022). As an anionic biopolymer, alginate exhibits better mucoadhesive properties than other biopolymers. It forms hydrogen bonds with mucin‐like glycoproteins (Shtenberg et al. 2018). Owing to their mucoadhesive and bioadhesive nature, they increase the residence time of drugs in different mucosal tissues, regulate drug release, and hence enhance bioavailability (Sosnik 2014; Chia et al. 2022).

In addition, okra gum is a plant‐derived polysaccharide extracted from immature and delicate fruits okra ( *Hibiscus esculentus* ) (Nayak et al. 2018). It is obtained from fresh or dried okra fruit and is composed of a polysaccharide consisting of galactose, rhamnose, galacturonic acid, and amino acids (Alamri et al. 2012; Archana et al. 2013; Kontogiorgos et al. 2012; Sinha, Ubaidulla, and Nayak 2015). Okra gum is a chemically inert, biodegradable, and biocompatible biopolymer of natural origin (Sinha, Ubaidulla, and Nayak 2015; Ghumman et al. 2018; Hussain et al. 2017; Patel, Baj, and Synthesis 2018). When extracted in water, okra gum produces a slimy, high‐viscosity solution. Owing to its high viscosity, this solution has high potential to be used as a mucoadhesive polymer in sustained‐release formulations (Zaharuddin, Noordin, and Kadivar 2014). Nevertheless, okra gum has found application in industry as an emulsifier, stabilizer, and thickening agent. Its favorable aqueous solubility and rheological characteristics in aqueous environments render it a promising substance for diverse applications in the development of pharmaceutical dosage formulations (Farooq, Malviya, and Sharma 2013). Studies also showed that tablets coated with okra gum have better physicochemical and mechanical properties than the core tablets (Köse, Gümüş Işik, and Bayraktar 2021).

Since ancient times, traditional and medicinal plants such as *Curcuma longa* (curcumin), *Aleo vera* (anthraquinones, saponins), *Camelia sinensis* (tea polyphenols), *Matricaria chamomilla* (coumarins, essential oils), and lycopene containing fruits and vegetables (i.e., tomatoes, grapes) have been used in the treatments of OMLs reported by several in vivo and clinical studies (Bahare Salehi et al. 2019). Here in this study, one of the natural antioxidant sources used to combat with oxidative stress generated by the cancer chemotherapeutics leading to OMLs is Thyme essential oil (TEO). It is well‐known that TEO has significant potential for use in various fields and it exhibits antifungal, antiviral, antioxidant, and antibacterial properties arising from the major volatile components, thymol and carvacrol, which allow TEO to have a broad range of therapeutic applications (Adam Kowalczyk, Przychodna, and Sylwia Sopata 2020). Previous studies showed that TEO has been successfully encapsulated using different encapsulation techniques (Tomazelli Júnior et al. 2018; Marques et al. 2021; Benavides et al. 2016). Ansarifar and Moradinezhad investigated the encapsulation of thyme essential oil within zein fiber films using the electrospinning method for potential use in food packaging (Ansarifar and Moradinezhad 2022). Another study explored the encapsulation of thyme essential oil in chitosan nanoparticles to investigate their thermal stability and antioxidant activity and found that encapsulation of essential oils using biopolymer‐based carrier systems enhances their stability (Ghaderi Ghahfarokhi et al. 2016). In another study, thyme essential oil was encapsulated in casein and maltodextrin using the spray drying method (Radünz et al. 2020). In the present study, due to their abundance, high biocompatibility, and abovementioned advantageous mucoadhesive properties, both alginate and okra gum were selected to be the carrier components for microencapsulation of TEO through electrospray method.

There are studies investigating the combinatorial effects of plant extracts to increase the bioefficacy and antioxidant potential of the film formulations. Neagu et al. developed a film formulation using extracts from *Taraxaci folium* and *Matricariae flos*. The synergistic effect of these two extracts on the anti‐inflammatory response was investigated (Neagu et al. 2023). In another study, *Usnea barbata* extract was added to a hydroxypropyl methyl cellulose (HPMC) and PEG400‐based mucoadhesive film formulation. The study evaluated in vitro anticancer and antimicrobial activities and in vivo cytotoxicity of the mucoadhesive films (Popovici et al. 2022). In the present study, in addition to TEO, olive leaf extracts (OLE) are aimed to be incorporated for developing mucoadhesive films. In a previous work from our research group, Köse et al. utilized carboxymethyl chitosan as a carrier system and incorporated olive leaf extract to develop a biopolymer film with enhanced bioactivity. It is well‐known that olive leaves are rich in phenolic compounds such as oleuropein, rutin, luteolin‐7‐glucoside, hydroxytyrosol, tyrosol, and verbacoside, which are associated with widely recognized and remarkable bioactivity of olive leaves, including their demonstrated antibacterial, antioxidant, antidiabetic, and anti‐inflammatory properties (Markhali, Teixeira, and Rocha 2020). Köse et al. also confirmed this with their findings indicating that conjugation of biopolymer films with olive leaf extracts successfully preserved the antioxidant properties (Köse, Gümüş Işik, and Bayraktar 2021). Moreover, Showraki et al. demonstrated that daily applications of OLE ointments have a healing effect on chemotherapy related‐oral mucositis in hamsters by decreasing mucosal inflammation and oxidative stress as suggested by the histopathology (Showraki et al. 2016).

**FIGURE 1:**
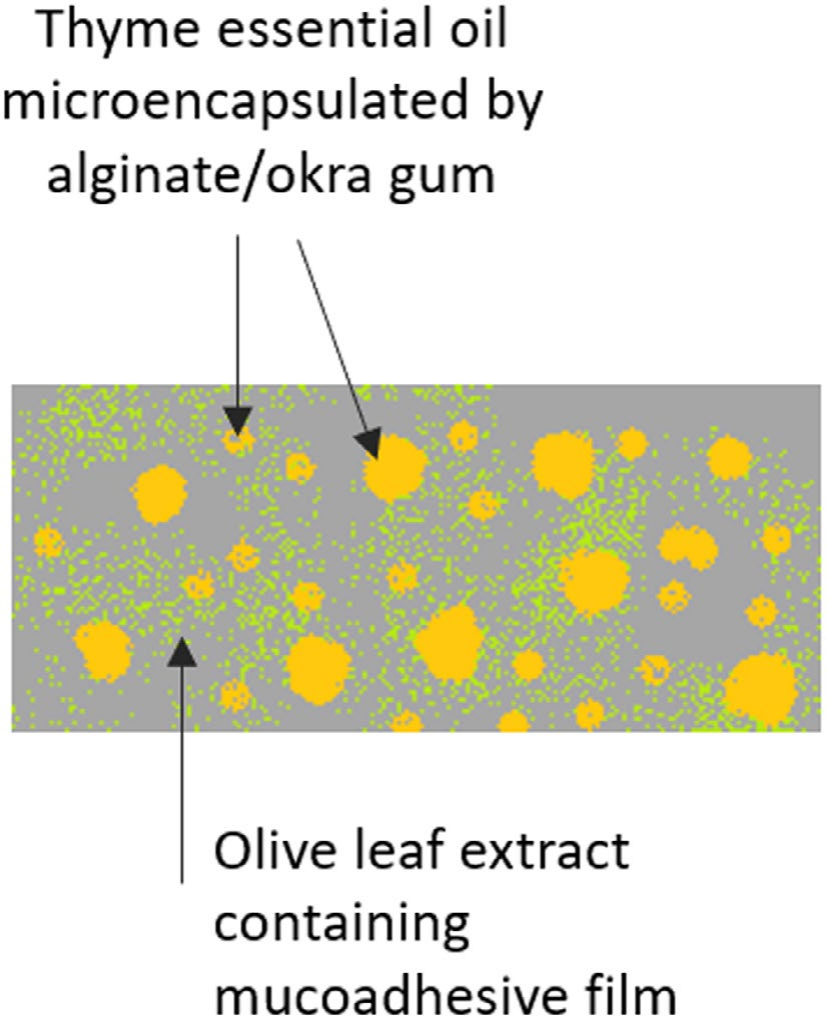
Microencapsulated TEO and OLE containing mucoadhesive film.

The general objective of this study, as illustrated in Figure 1, was to encapsulate TEO into sodium alginate/okra gum microcapsules in the mucoadhesive film formulation, where polymeric base is a bioadhesive multicomponent system composing of Carbopol934 (as gelling & stabilizing agent), HPMC (as binder & gelling agent), and PEG400 (hydrophilicity (release) controlling agent), containing OLE through solvent‐casting method at optimized conditions. It is clear that all of these compounds and materials exhibit certain advantageous and benefits in the delivery of therapeutics. Although they have been widely used in formulations developed based on various drug delivery strategies, there is no work investigating the combinatorial effects of all these compounds and materials in the form of mucoadhesive films, to the best of our knowledge. Therefore, in this study, a novel and innovative formulation was designed by combining microencapsulation and mucoadhesive film‐making techniques through separate optimizations based on Box–Behnken experimental design, and the resulting films were expected to demonstrate enhanced bioactivities.

## 2 Materials and Methods

### 2.1 Materials

Sodium alginate ((C_6_H_7_O_6_Na)_n_, MW > 200,000, CAS: 9005‐38‐3) and calcium chloride anhydrous (> 96%, MW: 110.99, CAS:10043‐52‐4) were purchased from Carlo Erba Reagents. Phosphate‐buffered saline (PBS powder, pH: 7.2–7.6, Product no: 76371–734), Carbopol 934 (powder, pH: 2.5–3.0, CAS: 9003‐01‐4), hydroxypropyl methylcellulose (HPMC, powder, CAS: 9004‐65‐3), and polyethylene glycol 400 (PEG400, av. MW: 380–420, CAS: 25322–68‐3) were purchased from VWR Science, Serva, Sigma Aldrich, and Merck, respectively. TEO was obtained from the local market while carvacrol (purified, > 97%) and thymol (purified, > 99%) standards used in GC–MS analyzes were purchased from Fluka, Buchs, Switzerland. Okra fruits and olive leaves were locally obtained. Cow buccal mucosa was gifted by a local butcher. All chemicals were used as it is.

### 2.2 Extraction of Olive Leaf

In the extraction and characterization of olive leaves, an optimized method reported by Bayçin et al. (2007) was used. For this, a certain amount of fresh olive leaves was mixed with 70% (by vol) aqueous ethanol solution for 24 h at 450 rpm and 25°C; then, the alcohol was removed using rotary evaporator (Selecta, RS‐3000 V); and the resulting aqueous extract was lyophilized using LyoQuest Laboratory Freeze‐dryer. The extraction efficiency was found to be 27.9%. The powdered OLE was kept under dark at room temperature for further analysis and until it is used. Total antioxidant capacity, total phenolic content, and oleuropein content of the extract were determined by various ways. A detailed explanation for the techniques used to characterize OLE including HPLC and FTIR analysis, and also, the results obtained are provided in Figures S1 and S2.

### 2.3 Extraction of Okra Gum

Okra Gum was obtained based on the method reported by Sinha, Ubaidulla, and Nayak (2015) with some modifications. In total, 10 g of ground and dried okra was soaked in distilled water at an S/L ratio of 1:50 at 25°C for 24 h. A total of 430 mL of liquid okra gum extract were obtained through filtration. The liquid extract was then mixed with acetone at a volume ratio of 1:3 to precipitate out the okra gum. The final precipitate was kept in an oven to dryness at 40°C. At the end, a total of 1.472 g of powdered okra gum (yield for acetone extraction was 14.6%) were obtained.

### 2.4 Characterization of Thyme Essential Oil

Locally obtained TEO was characterized through GC–MS analysis. Details of the analysis and the instrumentation used were given in Appendix S1. Results confirmed that TEO has two major components: 42.5% thymol and 41.5% carvacrol. The remaining 16% consists of 74 other compounds, most of which have mass fractions lower than 0.3%. The resulting GC chromatograms of thymol and carvacrol standards and TEO are given in Figure S3 while a full list of identified peaks is provided in Table S1.

### 2.5 Optimization of Electrospray Method for Fabrication of Microcapsules

TEO‐containing sodium alginate/okra gum microcapsules were fabricated using a laboratory‐type electrospray system with a single nozzle, for which a flow schematic is given in Figure S4. In this method, an emulsion solution containing certain amounts of TEO, sodium alginate and okra gum was first prepared and fed into the nozzle of the electrospray equipment via the help of a syringe pump. The electrospray system consists of a grounded collector plate, a high voltage power supply connected between the nozzle and the collecting plate. Since there are a number of parameters affecting the encapsulation yield, efficiency as well as the final morphology and size of the microcapsules, the optimum conditions for fabrication were determined through three parameters, three‐level centered Box–Behnken design method using Design Expert Version 7.0.0 (Stat‐Ease Inc. Minneapolis, MN, USA). This resulted in a total of 15 experiments where (a) flow rate of emulsion solution (mL/h), (b) applied voltage (potential difference, V), and (c) the distance between nozzle and collecting plate (cm) were varied. The levels of all three parameters used in experimental design are also listed in Table S2.

Morphology and average diameters of the microcapsules were determined by SZX16 Stereo Zoom Optical Microscope. The encapsulation efficiency was calculated as the ratio of the weight of the microcapsules obtained at the end of the process to the weight of the materials used initially, including the amount of essential oil. Encapsulation yield was calculated as the ratio of the amount of essential oil to be initially encapsulated for the process to the amount of essential oil trapped in the final particle.

### 2.6 Scanning Electron Microscopy (SEM) Analysis for the Optimized Microcapsules

SEM analysis was also performed to characterize the morphology and size of microcapsules obtained at the optimized electrospray conditions. Before the analysis, samples were coated with a thin gold layer under argon gas. Using Scanning Electron Microscope (Philips XL30 SFEG; FEI Company, Oregon, USA) and Quanta FL ESEM units, morphology of the microcapsules before and after release studies were examined.

### 2.7 Optimization of Mucoadhesive Film Formulation and Inclusion of Olive Leaf Extract (OLE)

In this study, solutions of three different polymers, which are Carbopol 934, hydroxypropyl methylcellulose (HPMC) and polyethylene glycol (PEG400), were used as film components at different mixing ratios. To optimize the structural and mechanical behavior of the resulting films, a three‐variable, 3‐level centered design method was again performed. According to the experimental design, a total of 27 formulations were prepared and the full list showing the mixing ratios in each formulation is provided in Table S3. In preparation, first a certain amount of Carbopol 934 required for the formulation was soaked in 1/3 of distilled water and left overnight to swell. At the end of 24 h, pH of the Carbopol 934 solution was adjusted to pH 6.0 using 2 M of NaOH and an immediate gel formation occurred. HPMC solution was prepared in the remaining portion of water as required by each formulation and left in the refrigerator for 24 h. Next, HPMC solution was added onto the Carbopol 934 gel and well‐mixed until a homogeneous solution is obtained. Finally, PEG was added to the solution to obtain the final gel formulation and left under mixing for another 24 h to equilibrate. In total, 30 mL of the resulting formulations was cast into petri dishes and dried at 60°C for 24 h. pH, viscosity as well as dispersion, mass uniformity, and thickness properties of the formulations were then investigated.

Inclusion of OLE was only performed using the optimum film formulations. For this, the same procedure was followed until PEG additions because various amounts of OLE (at concentrations of 5.0, 3.0, 1.0, 0.75, and 0.5 g/100 mL solution) were first dispersed in PEG to preserve the homogeneity. OLE containing mixtures were then poured into petri dishes and dried at 60°C for 24 h. UV analysis was employed to determine the OLE content in film formulations prepared with different concentrations of OLE. Briefly, OLE‐containing films were cut into 18 × 18 mm sections and dissolved in 10 mL of distilled water. The content uniformity was then examined using a UV‐spectrophotometer.

### 2.8 Addition of Thyme Essential Oil Microcapsules to OLE‐Containing Mucoadhesive Film Structure

To obtain the final OLE containing mucoadhesive films carrying TEO microcapsules, the OLE‐containing film solution prepared was first poured into a 10 mL petri dish and dried. Next, different concentrations (250, 500, 1000, 2000 mg in 100 mL of film solution) of TEO microcapsules were prepared in film solutions, and 10 mL of these solutions was poured onto the dried‐first layer of the film. Last, another 10 mL of OLE‐containing film solution was poured at the top layer and dried. The obtained films were then further characterized in terms of their structural, mechanical, and mucoadhesive properties.

### 2.9 Characterization of the Mucoadhesive Films for Optimization

#### 2.9.1 pH

pH measurements were carried out for all 27 samples in their solution and film forms using Eutech Instruments, pH 700 pH meter. For the films, samples with 18 × 18 mm dimensions were cut and dissolved in 10 mL of distilled water. Then, pH measurements were carried out for each sample.

#### 2.9.2 Viscosity

The viscosities of the prepared formulations at room temperature were measured using a rotational viscometer (J.P Selecta, ST 2020R), where calibration was performed using standard mineral oils with different viscosities.

#### 2.9.3 Mass Uniformity and Thickness

Mass uniformity and thickness measurements were performed using high precision scales and micrometers, respectively.

#### 2.9.4 Dispersion Test

Dispersion tests were carried out to determine the disintegration time for the films. For this, 2 mL of water was taken into a petri dish and the film of 18 × 18 mm size was placed onto the surface of water. The moment the film was completely dispersed was recorded as the disintegration time (Garsuch 2009; Garsuch and Breitkreutz 2010).

#### 2.9.5 Tensile Strength Measurement

The films were cut into suitable sizes for the test machine (Shimadzu, EHF‐LV020K2‐020 with 20 kN of dynamic capacity). During the test, the applied force and the strain were recorded. The tensile strength was then calculated using the following equation where the force value seen at the time of rupture of the film was recorded as the applied force.

(1) Tensile Strength=Applied force/Area of measurement

#### 2.9.6 Film Swelling and Abrasion Characteristics

Evaluations were made on the percentage of hydration and matrix erosion. The tested films were weighed (*M*_1_) and exposed to artificial saliva for a total of 100 s with 10 s of increments. At the end, the films were removed, excess liquid was washed out, and the final weight of the film was recorded (*M*_2_). After the films were dried in the oven, they were kept in the desiccator for 24 h and film weight was measured again (*M*_3_). The hydration and abrasion percentages were determined using the following equations, respectively.

(2) $$\%\text{Hydration}=\left(M_{2}-M_{1}\right)/M_{2}\times 100$$

(3) $$\%\text{Abrasion}=\left(M_{1}-M_{3}\right)/M_{1}\times 100$$

#### 2.9.7 Folding Endurance

Folding endurance was evaluated by counting the number of times the film could be folded without breaking.

#### 2.9.8 Contact Angle

Contact angles were determined using drop shape analysis. At room temperature, an optical contact angle meter (Drop Shape Analysis System DSA100, Krüss, Hamburg, Germany) was used to measure time‐dependent contact angles. A 7.5 μL drop of distilled water was dropped onto the film that was laying planar on the surface. After 10 s, the contact angle was calculated through the software (Drop Shape Analysis DSA1 v 1.90, Hamburg, Germany) (Garsuch and Breitkreutz 2010).

#### 2.9.9 In Vitro Mucoadhesion Time

The in vitro mucoadhesion time was determined by placing the films on cow buccal mucosa that was attached to the interior surface of a beaker using cyanoacrylate glue. Each film was 4 cm^2^ in size. Each film was wetted with 50 μL of artificial saliva before being placed to the cow buccal mucosa with gentle pressure for 20 s. The artificial saliva was kept at 37°C in a beaker containing 600 mL. After 2 min, the solution was agitated at 150 rpm to imitate the buccal cavity environment, and film adhesion was monitored for 24 h (Zhang et al. 2019).

#### 2.9.10 Ex Vivo Mucoadhesion Force

Using the abovementioned cow buccal mucosa, ex vivo adhesion strength was measured using a dynamometer. The films were cut into 4 cm^2^ pieces and placed on a support attached to the dynamometer with cyanoacrylate glue for mucoadhesive measurements. A piece of cow buccal mucosa was glued on a vessel's bottom. The free side of the film was wetted with simulated saliva fluid and applied to the cow buccal mucosa with light pressure for 20 s using fingertip. The vessel was filled with simulated saliva fluid, and the measurement began after 2 min. The average of three measurements yields the highest adhesive force (Perioli et al. 2004). All these abovementioned tests were replicated for the optimized film formulation, and results were given with standard deviations.

#### 2.9.11 Antioxidant Analysis

The total antioxidant capacity of the final optimized film formulation was determined using the same ABTS based method explained in Appendix S1 for the determination of antioxidant capacity of olive leaf extract.

#### 2.9.12 Cytotoxicity Analysis

As required by ISO 10993, both sides of the final optimized film were sterilized with UV light for 45 min. They were then placed in a serum‐free culture medium at a concentration of 6 cm^2^/mL, and the extraction process was carried out for 24 h at 37°C. For the cytotoxicity test, the L929 mouse fibroblast cell line was used. In a humidified incubator at 37°C with 5% CO_2_, the cells were cultivated in a T75 flask containing DMEM‐High Glucose, 10% FBS (Fetal Bovine Serum), 1% l‐glutamine, and 0.1% Penicillin/Streptomycin. When the cells achieved 80% confluence, the experiment was initiated. P41 cells were employed in the study. A 96‐well culture plate was used to seed the cells, with a concentration of 10^5^ cells/mL. The cells were cultivated for 24 h at 37°C with 5% CO_2_ in a humidified incubator before the extract were applied.

The cell viability assay for Alamar Blue was utilized in the cytotoxicity analysis. The Alamar Blue test is a viability assay in which live cells interact with resazurin dye to change the medium's color from blue to pink. It is possible to monitor the same cells for up to 3 days with this test since it does not damage the cells or impair their viability. The cells were exposed to 1, 1/5, 1/10, 1/50, and 1/100 dilutions of the film extract. These dilutions were UV‐sterilized in preparation for the film. The cells were kept in their own culture medium in the control group (without film samples). Following a 24, 48, and 72 h applications of the extract, the cells' media were taken out and replaced with a serum‐free medium containing 10% Alamar Blue. After that, the cells were incubated for 4 h at 37°C, 5% CO_2_, and in a humidified incubator. After incubation, a spectrophotometer set to measure color changes between 570 and 600 nm was used.

#### 2.9.13 Antiviral, Antibacterial, and Antifungal Activity Analyses

The antiviral, antibacterial, and antifungal activity analyses of the final product were performed by accredited laboratories based on the standard method TS EN 14476 + A1:2019‐09. According to this standard, films were completely dissolved in distilled water and the sample for analysis was prepared at a concentration of 80% by vol. Bacterial tests were conducted using four different bacterial strains, *E. coli* , *S. aureus* , *P. aureginosa*, and *E. hirae* , for which the contact time was 1‐min. *Poliovirus Type 1* and *M. Novovirus* were used in virucidal analysis while fungicidal tests were done against *C. albicans* and *A. brasiliensis* species. The contact time in virucidal and fungicidal analyses were 2 min and 15 min, respectively. Bovine serum albumin solution at a concentration of 3 g/L was used as inhibitor and a clean solution for all analysis.

## 3 Results and Discussion

### 3.1 Investigation of the Effect of Okra Gum Concentration on the Emulsion Stability of Thyme Essential Oil (TEO)

**FIGURE 2:**
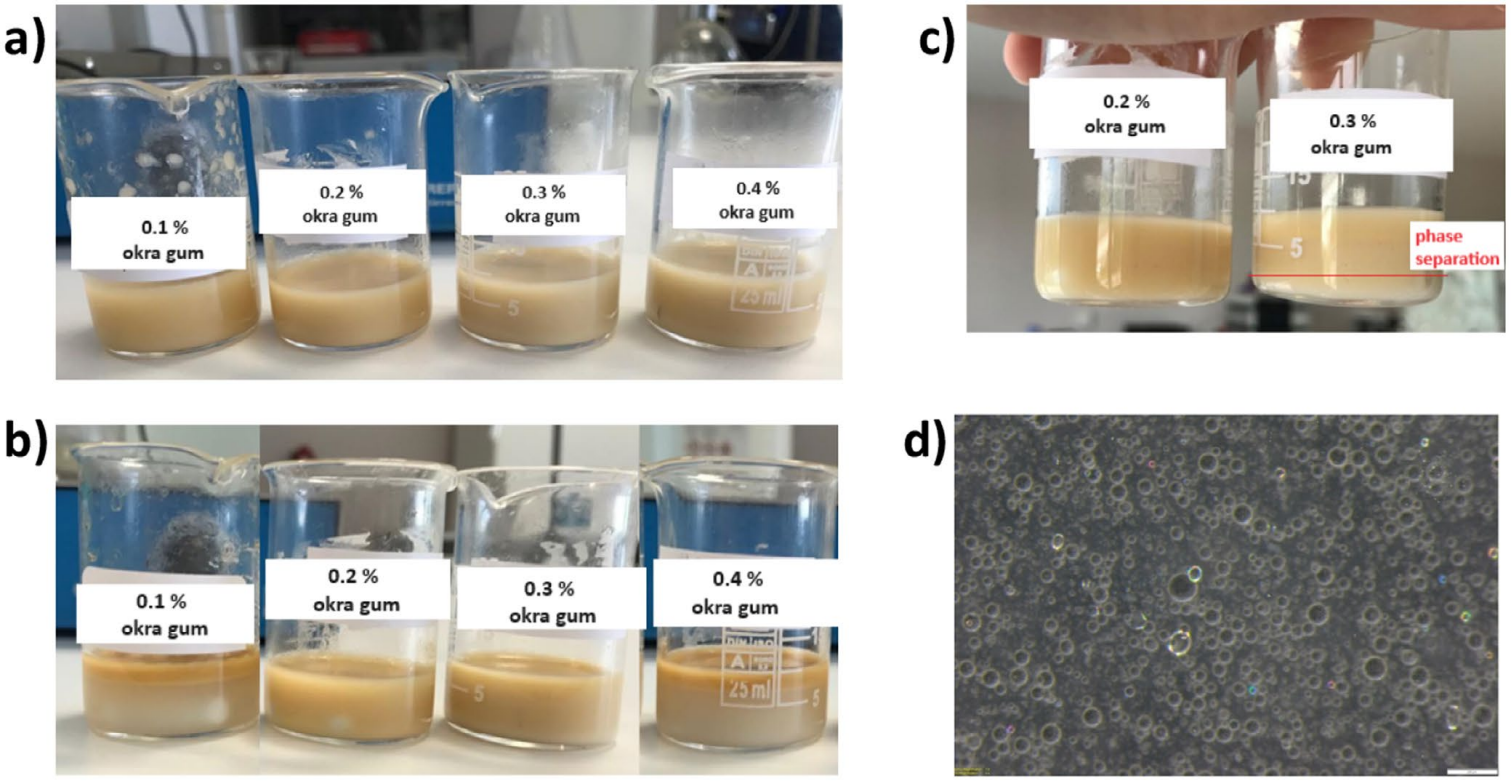
(a) Emulsions at the time of preparation (*t* = 0), (b) Emulsions after 24 h (*t* = 24 h), (c) Close‐up image for emulsions containing 0.2% and 0.3% okra gum after 24 h for the ease of comparison, and (d). Microscope image of the most stable emulsion containing 0.2% okra gum after 24 h. Scale bar in d is 200 μm. Photograph courtesy of “Kubra Goktas.” Copyright 2023.

Prior to optimization of microencapsulation parameters, the effect of okra gum concentration was investigated on the feeding emulsion solution containing TEO. For this, 10 mL of four different emulsions was prepared with okra gum concentrations of 0.1%, 0.2%, 0.3%, and 0.4% while TEO and sodium alginate ratios were kept constant at 15% and 1.0%, respectively. All four emulsion solutions were analyzed visually and using optical microscope at the time of preparation as well as after 24 h. Figure 2 shows the images of these emulsion solutions taken at *t* = 0 and after 1 day for comparison.

As shown in Figure 2a, all four emulsions containing different concentrations of okra gum initially appeared to be homogeneous. However, after 24 h, a clear phase separation was observed, and the emulsion stabilities were not remained for the samples (Figure 2b,c) except the one containing 0.2% okra gum. From microscopic analysis (Figure 2d), TEO droplets were homogeneously distributed, and hence, emulsion with 0.2% okra gum appeared to be the most homogeneous and stable after 1‐day period.

### 3.2 Optimization of Microencapsulation Parameters

A laboratory‐type electrospray system was used to encapsulate TEO into alginate/okra gum. The composition of feeding emulsion solution containing both TEO and alginate/okra gum were determined through emulsion stabilization experiments as essential oil (15%), sodium alginate (1.0%), and okra gum (0.2%). However, for such electrospray method, there are other significant parameters such as flow rate of feeding solution, potential difference applied and distance between the sample container's nozzle and collector plate, which may affect encapsulation efficiency and yield as well as the particle size of the microcapsules. Therefore, a multiparameter, three‐level centered Box–Behnken method was used to design the microencapsulation experiments and hence, to optimize the electrospray parameters. The design table for a total of 15 experiments was given in Table 1 showing the selected parameters with their levels of examination and the corresponding encapsulation responses of interest.

**TABLE 1:** Experimental design parameters for microencapsulation of TEO and the corresponding responses as encapsulation efficiency, average microcapsule diameter, and encapsulation yield.

Run	*A*. Flow rate (mL/h)	*B*. Potential difference (kV)	*C*. Distance (cm)	*Y*_1_. Encapsulation efficiency (%)	*Y*_2_. Average microcapsule diameter (mm)	*Y*_3_. Encapsulation yield (%)
1	0.5	10	10	94.08	0.79	41.42
2	2.5	10	10	95.52	0.68	74.95
3	0.5	14	10	59.68	0.63	20.78
4	2.5	14	10	40.94	0.56	31.08
5	0.5	12	8	32.64	0.60	16.45
6	2.5	12	8	72.96	0.57	58.28
7	0.5	12	12	87.04	0.69	30.92
8	2.5	12	12	50.58	0.63	41.55
9	1.5	10	8	99.76	0.62	70.78
10	1.5	14	8	16.05	0.41	12.07
11	1.5	10	12	97.85	0.59	68.04
12	1.5	14	12	95.15	0.51	69.98
13	1.5	12	10	85.95	0.56	61.59
14	1.5	12	10	87.09	0.55	64.06
15	1.5	12	10	83.17	0.55	63.33

All results given in Table 1 were statistically investigated using Design Expert to obtain the models of best fit for each response. According to the calculated standard deviation, *R* ^2^, and adjusted *R* ^2^ values for each response, the reduced cubic model was selected to be the best performing and the resulting design equations were given in the following.

$$\begin{array}{cc} \text{Encapsulation Efficiency}\;\left(\%\right)=\; & +85.40-21.60B+19.30C-5.04\mathrm{AB}-19.19\mathrm{AC}\\ & +20.52\mathrm{BC}-14.62A^{2}-9.98C^{2}-11.29A^{2}C \end{array}$$

$$\begin{array}{cc} \text{Average Microcapsule Diameter}\;\left(\mathrm{mm}\right)=\; & +0.555-0.215A-0.0723B+0.0333\mathrm{BC}\\ & +0.1009A^{2}-9.98C^{2}-11.29A^{2}C \end{array}$$

$$\begin{array}{c} \text{Encapsulation yield}\;\left(\%\right)=+62.99+13.11A-14.19B+13.79C-5.81\mathrm{AB}-7.80\mathrm{AC}\\ +15.16\mathrm{BC}-19.68A^{2}-1.26B^{2}-6.52C^{2}-1.94A^{2}B-14.36A^{2}C-2.16AB^{2} \end{array}$$

The subsequent analysis of variance (ANOVA) results were obtained for each response separately. Table 2 summarizes the results of ANOVA performed for encapsulation efficiency (*Y*_1_), while those obtained for average microcapsule diameter and encapsulation yield are provided in Tables S4 and S5, respectively.

**TABLE 2:** Results of ANOVA for encapsulation efficiency (*R* ^2^: 0.9992; adjusted *R* ^2^: 0.9943).

Source	Sum of squares	df	Mean square	*F*	*p*	
Model	10013.77	12	834.48	205.25	0.0049	Significant
*A*. Flow rate	3.72	1	3.72	0.9162	0.4395	
*B*. Potential difference	1866.67	1	1866.67	459.12	0.0022	
*C*. Distance	1489.57	1	1489.57	366.37	0.0027	
*AB*	101.81	1	101.81	25.04	0.0377	
*AC*	1473.79	1	1473.79	362.49	0.0027	
*BC*	1640.66	1	1640.66	403.53	0.0025	
*A* ^2^	789.53	1	789.53	194.19	0.0051	
*B* ^2^	11.63	1	11.63	2.86	0.2329	
*C* ^2^	367.42	1	367.42	90.37	0.0109	
*A* ^2^ *B*	0.8256	1	0.8256	0.2031	0.6964	
*A* ^2^ *C*	255.04	1	255.04	62.73	0.0156	
*AB* ^2^	55.97	1	55.97	13.77	0.0656	
Pure error	8.13	2	4.07			
Cor total	10,021.90	14				

From Table 2, it can be seen that when single terms of the model were compared to each other, the variations in potential difference applied during encapsulation and distance between the nozzle of sample container and collector plate were found to have the most significant effects on the encapsulation efficiency due to their large *F*‐values and significantly small *p*‐values (< 0.005). On the other hand, flow rate of the emulsion demonstrated the least significant effect on encapsulation efficiency. As expected, the effect of emulsion flow rate became more significant when considering cross‐terms of flow rate and the spraying distance.

**FIGURE 3:**
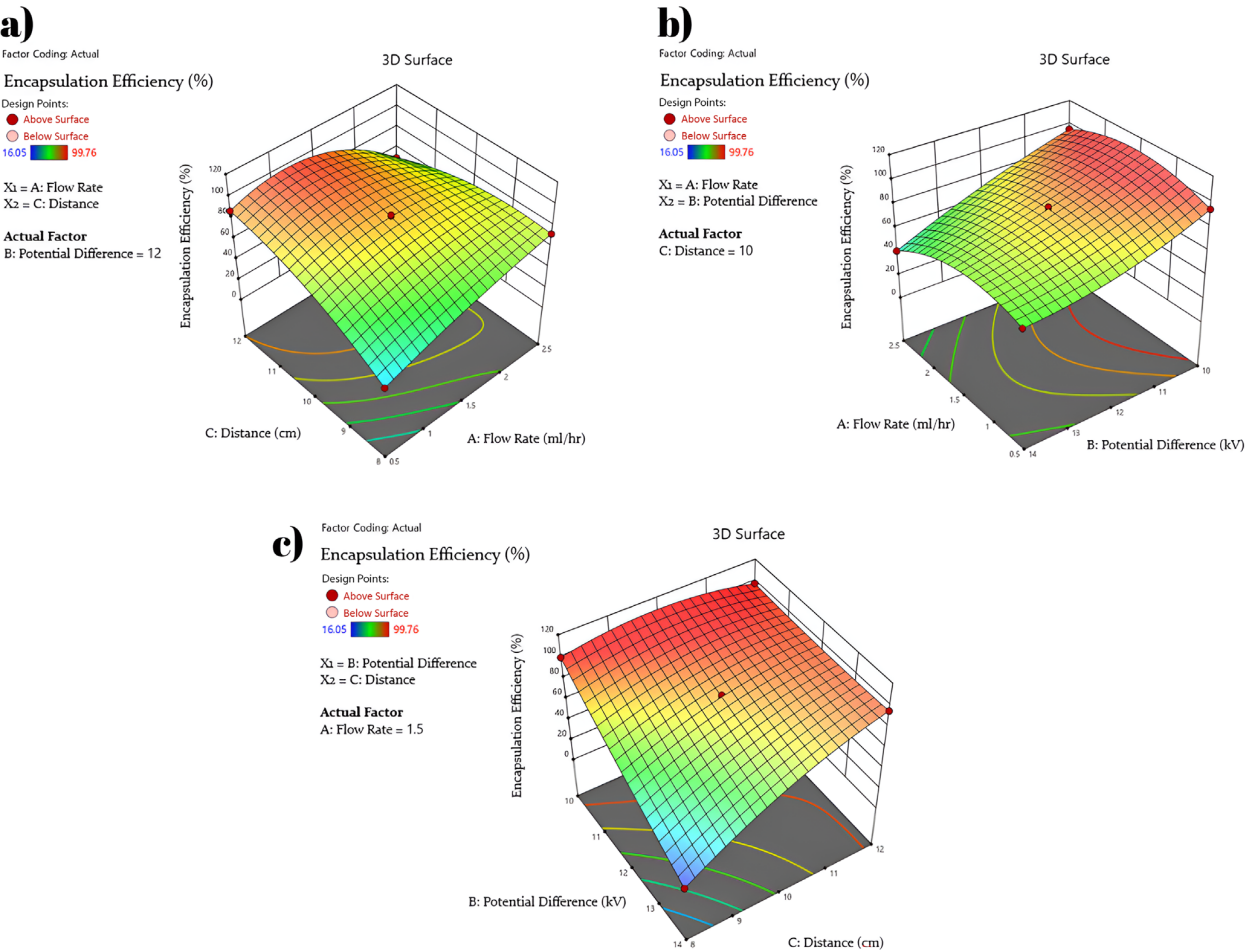
Response‐surface plots obtained for encapsulation efficiency based on binary relationships between variables; (a) Distance and Flow rate, (b) Flow rate and Potential difference, and (c) Potential difference and Distance.

The 3D response‐surface plots were also constructed to elucidate the binary relationships between variables with respect to the extraction efficiency and yield as well as the particle size. Figure 3 shows the 3D response‐surface plots obtained for encapsulation efficiency, while those for average microcapsule diameter and encapsulation yield are provided in Figures S5 and S6, respectively.

From Figure 3a, it is clear that at flow rates within a range of 0.5–1.5 mL/h and spraying distance from 10 to 12 cm, there is a greater likelihood to achieve an encapsulation efficiency greater than 90%. When binary effect of flow rate and potential difference were considered, a more significant effect of potential difference on the extraction efficiency was evident as shown in Figure 3b. The lower the potential difference provides the greater encapsulation efficiency regardless of the given range of flow rate. Figure 3c shows the dual effect of two of the most significant variables as determined by ANOVA results, and it was found that lowering potential difference while increasing the spraying distance results in a greater encapsulation efficiency.

**FIGURE 4:**
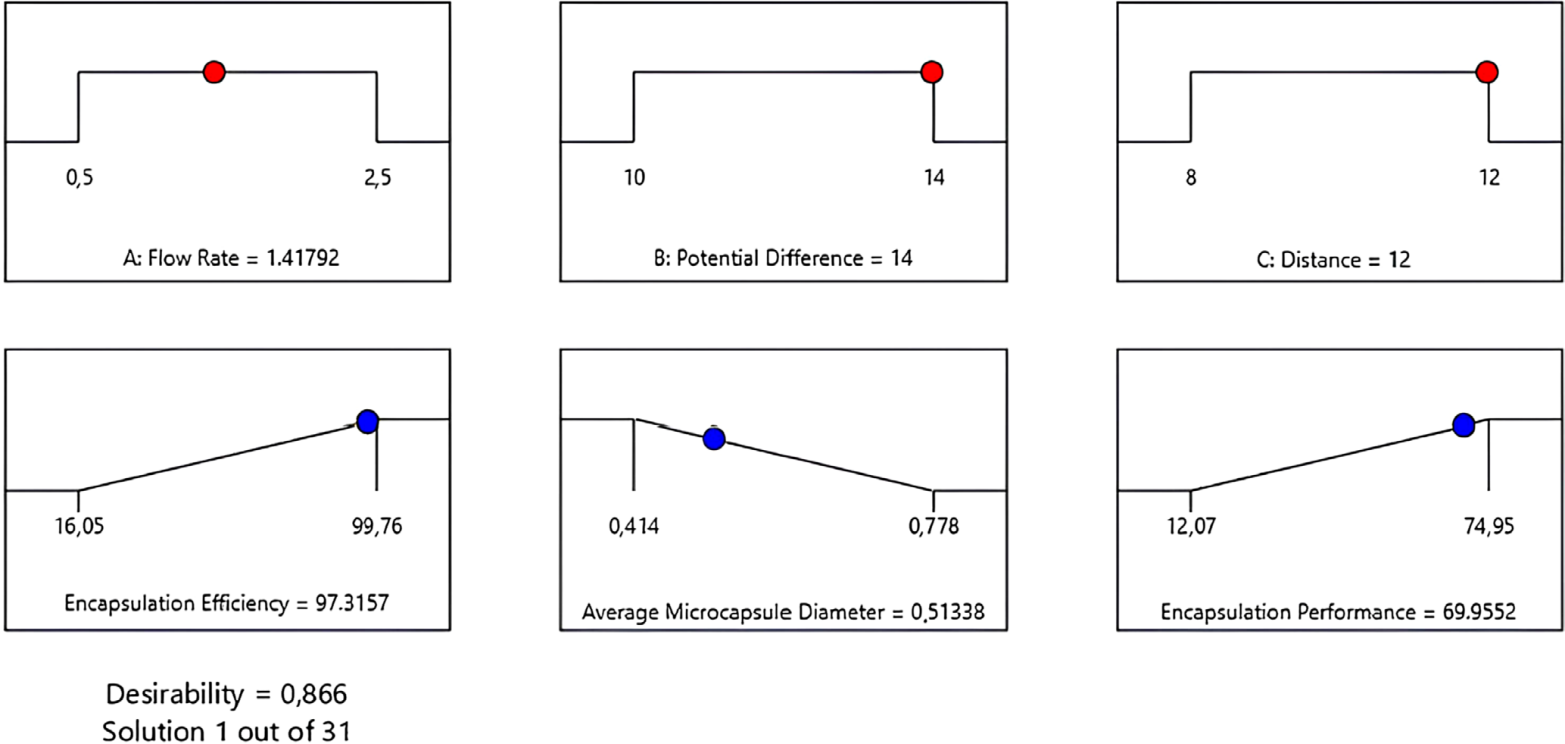
Ramp Graphs obtained by multiple optimizations to maximize encapsulation efficiency and yield, and to minimize average microcapsule diameter. (Desirability factor was determined as 0.87 for the optimized solution out of a total of 31 runs.)

3D response‐surface plots constructed for the average microcapsule diameter and encapsulation yield (Figures S5 and S6) demonstrated a similar trend regarding binary relationships of variables. According to these results, keeping the flow rate at low‐to‐mid range and decreasing the potential difference while increasing the spraying distance seem to have a positive effect to determine the optimum conditions. However, a simultaneous multiple optimizations were also applied to all data and the optimum values of all three parameters were determined as a flow rate of 1.42 mL/h, a potential difference of 14 kV, and a spraying distance of 12 cm, thereby the encapsulation efficiency and yield were maximized while the average microcapsule diameter was minimized, as shown by the ramp graphs given in Figure 4. At these conditions, based on the multiple optimizations, the average microcapsule diameter was found to be 0.513 mm, the encapsulation efficiency to be 97.32% and the encapsulation yield to be 69.96%.

### 3.3 Morphological Characterization of Microcapsules

**FIGURE 5:**
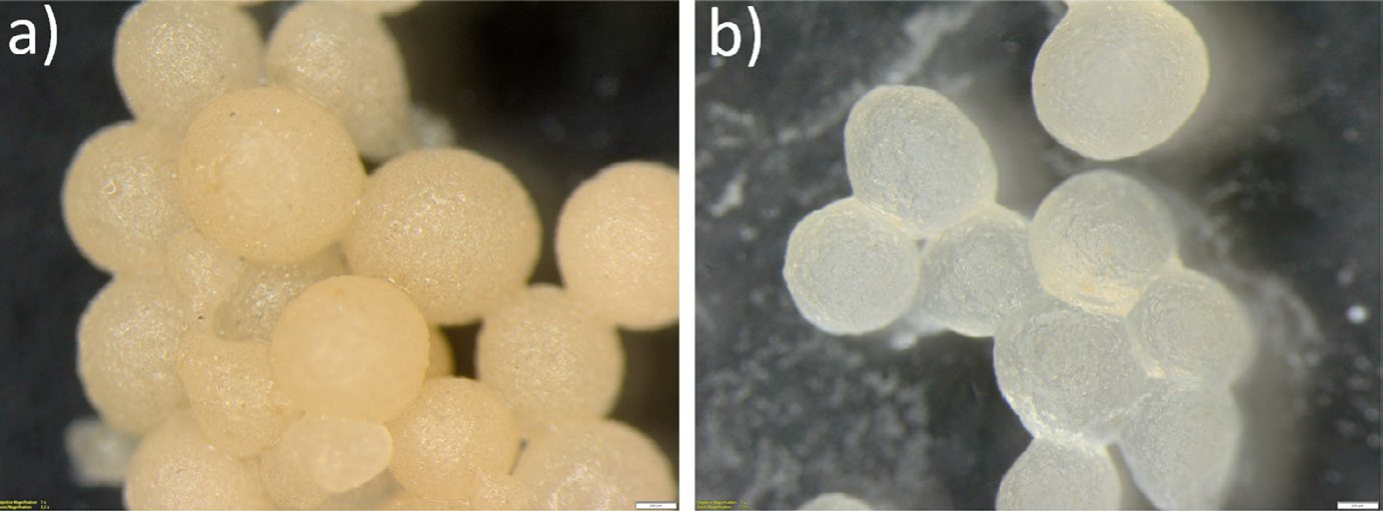
Optical microscope images of microcapsules containing TEO (a) before and (b) after the 24‐h release study. Scale bars are 200 μM.

At the optimized conditions, thyme oil encapsulation was performed, and the resulting microcapsules were characterized in terms of their morphology through SEM and optical microscope. Figure 5 shows the optical microscope images of microcapsules before and after the release, which was conducted using a 50 mL of phosphate buffer saline (PBS at pH = 7.4, 60% by vol.) and ethanol (40% by vol.) mixture at room temperature under gentle mixing for 24 h (Hosseini et al. 2013). Since the size of microcapsules was quite big, the SEM images obtained for the same samples are only provided in Figure S7, because they are difficult to interpret. However, the SEM data showed a clear deterioration upon mixing the microcapsules with PBS and ethanol mixture, confirming the release of all TEO content of microcapsules.

Microcapsules with an average diameter of 500 μm were found to initially have a clear light orange color due to the essential oil content, as shown in Figure 5a. Treating them in PBS + ethanol mixture for 24 h led to a severe structural deterioration according to the SEM analysis as shown in Figure S7, and a distinct color change from light orange to pale yellow was observed through optical microscopy (Figure 5b). In addition, in vitro release kinetics study was conducted to validate the encapsulation efficiency of the microcapsules and to ensure the reliability of the results. The data obtained were fitted to a release kinetic model, and it was found that release kinetics of both thymol and carvacrol are best described by the Korsmeyer‐Peppas model, with *R* ^2^ values of 0.902 and 0.896, respectively, ensuring that all TEO content is successfully released from microcapsules at the end of the 24 h‐time period. A detail graphical representations showing the release kinetics of TEO (wrt to thymol and carvacrol) according to the Korsmeyer‐Peppas model fitting are provided in Figure S8.

### 3.4 Optimization of Film Formulation

Prior to the inclusion of the OLE to custom‐designed mucoadhesive films, polymeric components of the films were first optimized through a multi‐variable, 3‐level Box–Behnken experimental design. This resulted in a total of 27 film formulations where Carbopol 934 (C 934), HPMC and PEG contents were varied at mixing ratios given in Table S3. The required amount of water was added to each formulation to complement the percentage. The following tests, gentle peeling off from the petri dish, pH, viscosity, dispersion, mass uniformity, and thickness measurements, were performed for all films. Results obtained for each formulation along with their polymer compositions were given in Table 3.

**TABLE 3:** Characterization results of mucoadhesive film formulations along with polymer composition.

Formula number	C 934 (w/v)	HPMC (w/v)	PEG (w/v)	Peel off from petri dish	Viscosity (Pa s)	Solution pH (before film formation)	Film pH^a^	Disintegration time (s)	Thickness (mm)	Weight (g)
F‐1	0.1	1.5	0.4	−	0.07	7.53	—	—	—	—
F‐2^b^	0.1	2.0	0.4	+	0.11	7.89	8.10	37.86	0.309	0.06
**F‐3**	**0.1**	**2.5**	**0.4**	+	**0.13**	**7.40**	**7.66**	**91.33**	**0.278**	**0.15**
F‐4	0.1	1.5	0.8	−	0.10	7.12	—	—	—	—
F‐5^b^	0.1	2.0	0.8	+	0.09	7.50	7.79	49.86	0.488	0.09
**F‐6**	**0.1**	**2.5**	**0.8**	+	**0.11**	**7.17**	**7.68**	**34.89**	**0.261**	**0.06**
F‐7	0.1	1.5	1.2	−	0.06	7.45	—	—	—	—
**F‐8**	**0.1**	**2.0**	**1.2**	+	**0.08**	**7.35**	**7.61**	**45.09**	**0.378**	**0.07**
**F‐9**	**0.1**	**2.5**	**1.2**	+	**0.09**	**7.38**	**7.63**	**73.42**	**0.247**	**0.07**
F‐10	0.15	1.5	0.4	−	0.18	7.53	—	—	—	—
F‐11^c^	0.15	2.0	0.4	+	0.40	7.31	7.35	11.83	0.442	0.03
**F‐12**	**0.15**	**2.5**	**0.4**	+	**0.50**	**7.38**	**7.61**	**34.41**	**0.236**	**0.05**
F‐13	0.15	1.5	0.8	−	0.36	7.43	—	—	—	—
F‐14	0.15	2.0	0.8	−	0.51	7.37	—	—	—	—
F‐15^b^	0.15	2.5	0.8	+	0.61	7.46	7.81	61.79	0.342	0.05
F‐16^b^	0.15	1.5	1.2	+	0.31	8.10	8.75	70.33	0.37	0.09
F‐17^b^	0.15	2.0	1.2	+	0.43	7.69	8.00	36.84	0.44	0.1
F‐18	0.15	2.5	1.2	−	0.33	7.47	—	—	—	—
F‐19	0.2	1.5	0.4	−	0.82	7.49	—	—	—	—
F‐20^b^	0.2	2.0	0.4	+	1.05	8.06	8.27	8.91	0.415	0.03
F‐21^b^	0.2	2.5	0.4	+	0.95	7.49	7.86	30.23	0.371	0.06
F‐22	0.2	1.5	0.8	−	0.67	7.14	—	—	—	—
F‐23	0.2	2.0	0.8	−	0.95	7.34	—	—	—	—
F‐24	0.2	2.5	0.8	−	0.93	7.56	—	—	—	—
F‐25	0.2	1.5	1.2	−	0.93	7.22	—	—	—	—
F‐26	0.2	2.0	1.2	−	0.92	7.48	—	—	—	—
F‐27	0.2	2.5	1.2	−	1.01	7.32	—	—	—	—

*Note:* Rows highlighted in gray show the films with inappropriate separation from the surface of Petri dishes. Bold values indicates the formulations which met the film pH criteria of 7.6 or approximately 7.6.

^a^ Film pH was measured by dissolving the film with a size of 18 × 18 mm in 10 mL of distilled water.

^b^ pH of the films was higher than 7.7 (approximate pH value of 7.6 is desirable for films).

^c^ Film exhibited a very low disintegration time.

**FIGURE 6:**
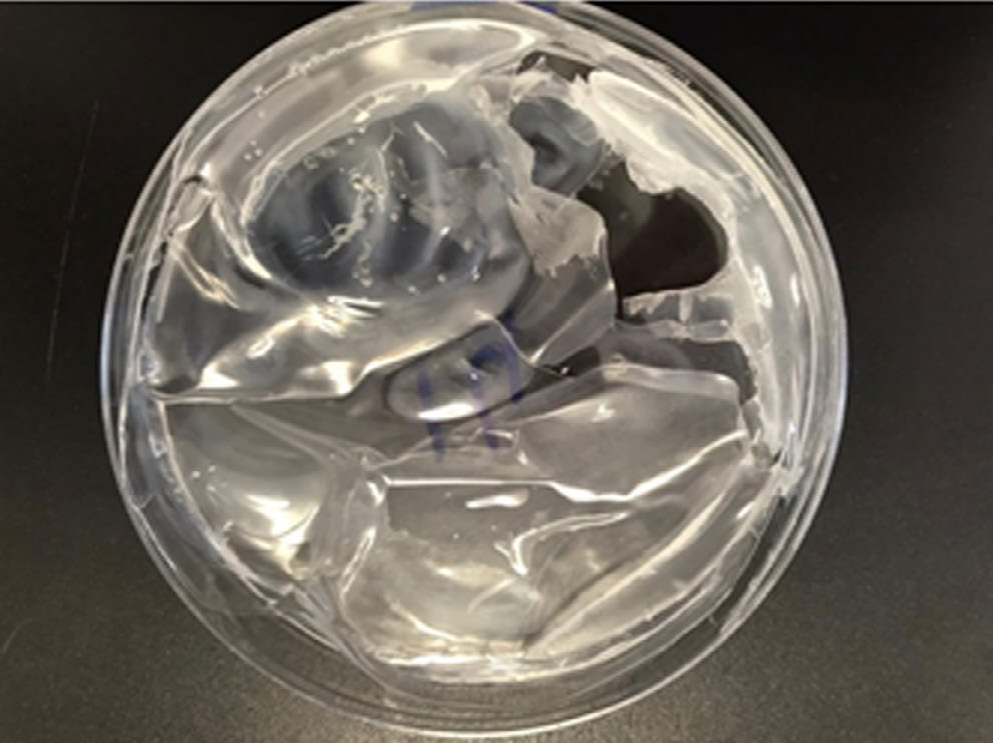
Representative image of deteriorating film structure of Formulation 14. Photograph courtesy of “Kubra Goktas.” Copyright 2023.

As seen in Table 3, 13 formulations out of the 27 showed a good separation from the Petri dishes preserving the film structure, while the remaining 14 formulations could not be well peeled off from the Petri dishes, a representative image of a deteriorating film structure observed for Formulation 14 (F‐14) was given in Figure 6. Due to this structural deterioration, further characterization associated with film properties were not conducted for those samples.

Since highly acidic or basic conditions may disrupt the mucosal structure, pH is a crucial parameter in determining the optimum film structure. It has been reported that the desirable pH range for a mucoadhesive film to be compatible with the oral mucosa is between 6.2 and 7.6 (Baliga, Muglikar, and Kale 2013). The other important criterion was solution viscosities, and it was observed that increasing C934 fraction in the formulations along with the other two polymers resulted in higher viscosity in solution. This made the removal of air bubbles harder, and hence, for the films with high viscosity, an unwanted bubbly structure was observed. Besides, a moderate or long disintegration time is highly desirable for a mucoadhesive film. Therefore, a threshold value of 30 s was determined as a selection criterion, films exhibited a disintegration time greater than 30 s were considered for further analysis. According to these, five formulations (F‐3, F‐6, F‐8, F‐9, and F‐12) in Table 3 were found to have successfully met the desired criteria. To assess the reproducibility of the selected films, experiments were repeated in triplicate, and weight and thickness measurements were conducted for all five formulations. These results are provided in Table S6, while results of the top 3 formulations based on the repeated tests on mass uniformity and thickness were given in Table 4.

**TABLE 4:** The top 3 formulations of mucoadhesive films.

Formula number	C 934 (w/v)	HPMC (w/v)	PEG (w/v)	Thickness (mm) ± SD	Weight (g) ± SD
F‐8	0.1	2.0	1.2	0.38 ± 0.05	0.07 ± 0.03
F‐9	0.1	2.5	1.2	0.25 ± 0.08	0.06 ± 0.02
F‐12	0.15	2.5	0.4	0.24 ± 0.09	0.05 ± 0.03

### 3.5 Determination of Optimum OLE Amount for Film Formulation

**FIGURE 7:**
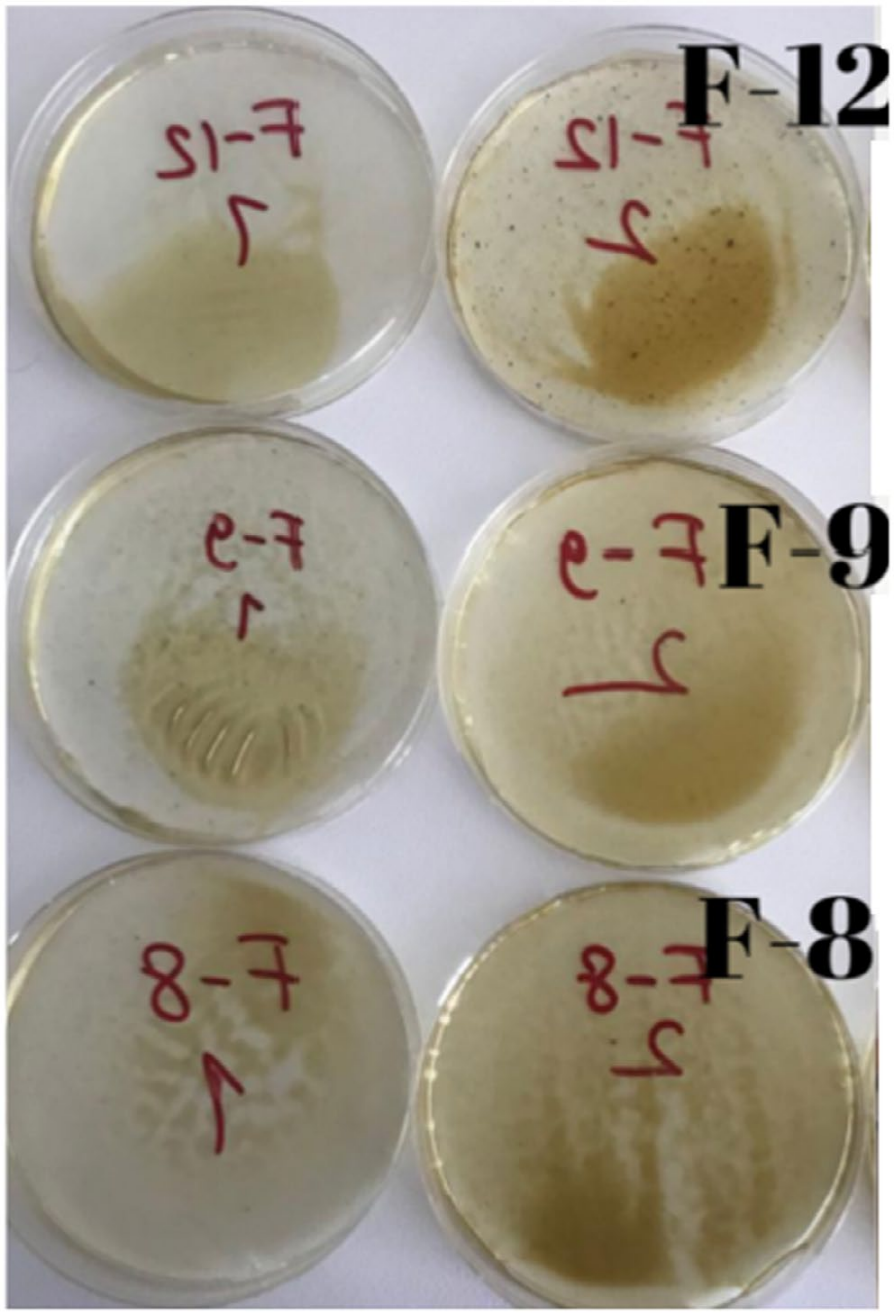
The selected three film formulations with 1% by w/v OLE content. Photograph courtesy of “Kubra Goktas.” Copyright 2023.

First, the most suitable formulation out of the three selected (F‐8, F‐9, and F‐12) for OLE inclusion was determined through examining the film‐making characteristics by adding an equal amount of OLE (1% w/v). Figure 7 shows the appearances of those films after drying, and in terms of flexibility, formulation 8 (F‐8) was found to be providing a better film structure and uniformity than the other two formulations.

**FIGURE 8:**
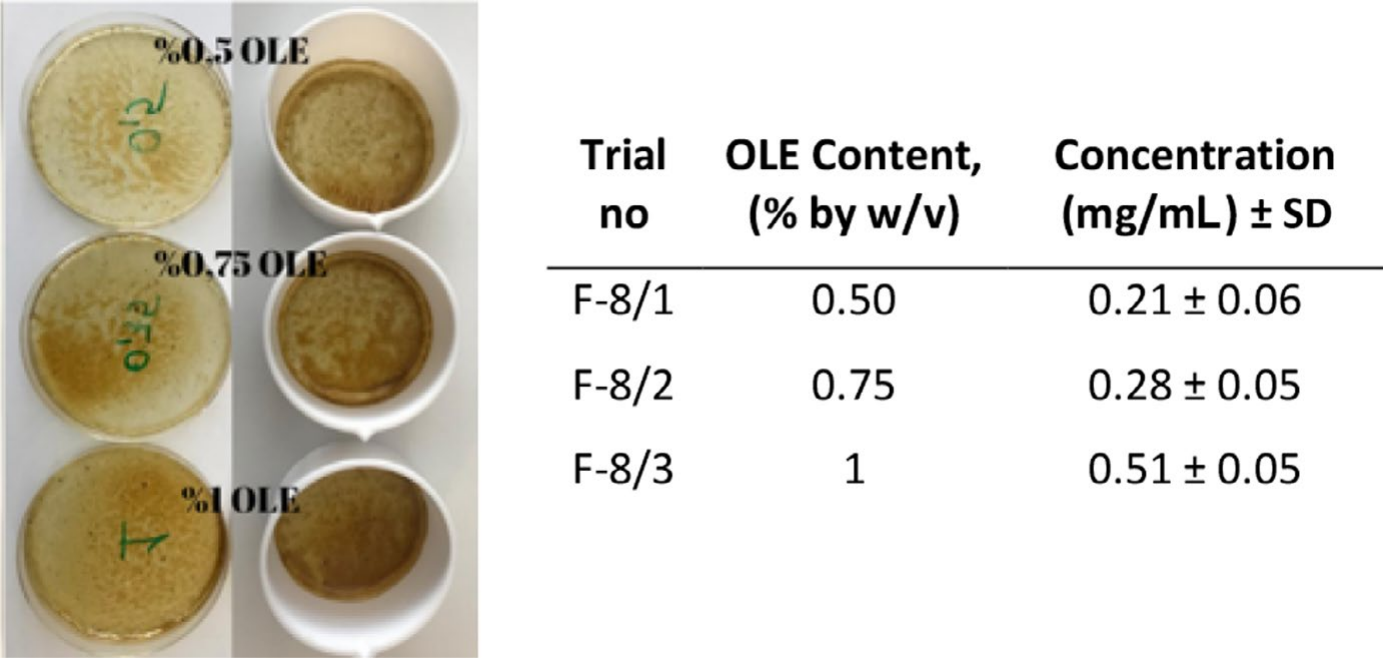
Mucoadhesive oral films containing different amounts of OLE (0.5%, 0.75%, and 1% by w/v). Inset table shows the OLE content of the films determined through UV‐spectrophotometer. Photograph courtesy of “Kubra Goktas.” Copyright 2023.

After that, different amounts of OLE (5, 3, 1, 0.75, 0.5 g OLE/100 mL) containing films made based on Formulation F‐8 were prepared to determine the OLE loading capacity of the films without damaging the uniformity. Films with the highest OLE loading (5% and 3%) demonstrated severe deformation when peeling off from petri dishes (Figure S9). As shown in Figure 8, films with lower OLE content maintained structural uniformity. These films were then cut to 18 × 18 mm and dissolved in 10 mL of pure water. The inset table in Figure 8 also shows the calculated OLE content determined through UV‐spectrophotometer. Consequently, since there was no difference in content uniformity and physical properties for the best performing films, the formulation with the highest OLE content (1% OLE by w/v) was chosen as optimum.

### 3.6 Loading of TEO Microcapsules on OLE‐Containing Mucoadhesive Films

Based on the optimization study, final mucoadhesive oral film samples were prepared according to the Formulation 8 (F‐8), which also include 1% OLE by w/v. These films were then loaded with TEO microcapsules at ratios of 0.25% (250 mg dry microcapsules in 100 mL film solution), 0.5% (500 mg), 1% (1000 mg), and 2% (2000 mg). It must be noted that dry powdered microcapsules contain 15% of essential oil encapsulated into sodium alginate/okra gum as described in the previous sections.

**FIGURE 9:**
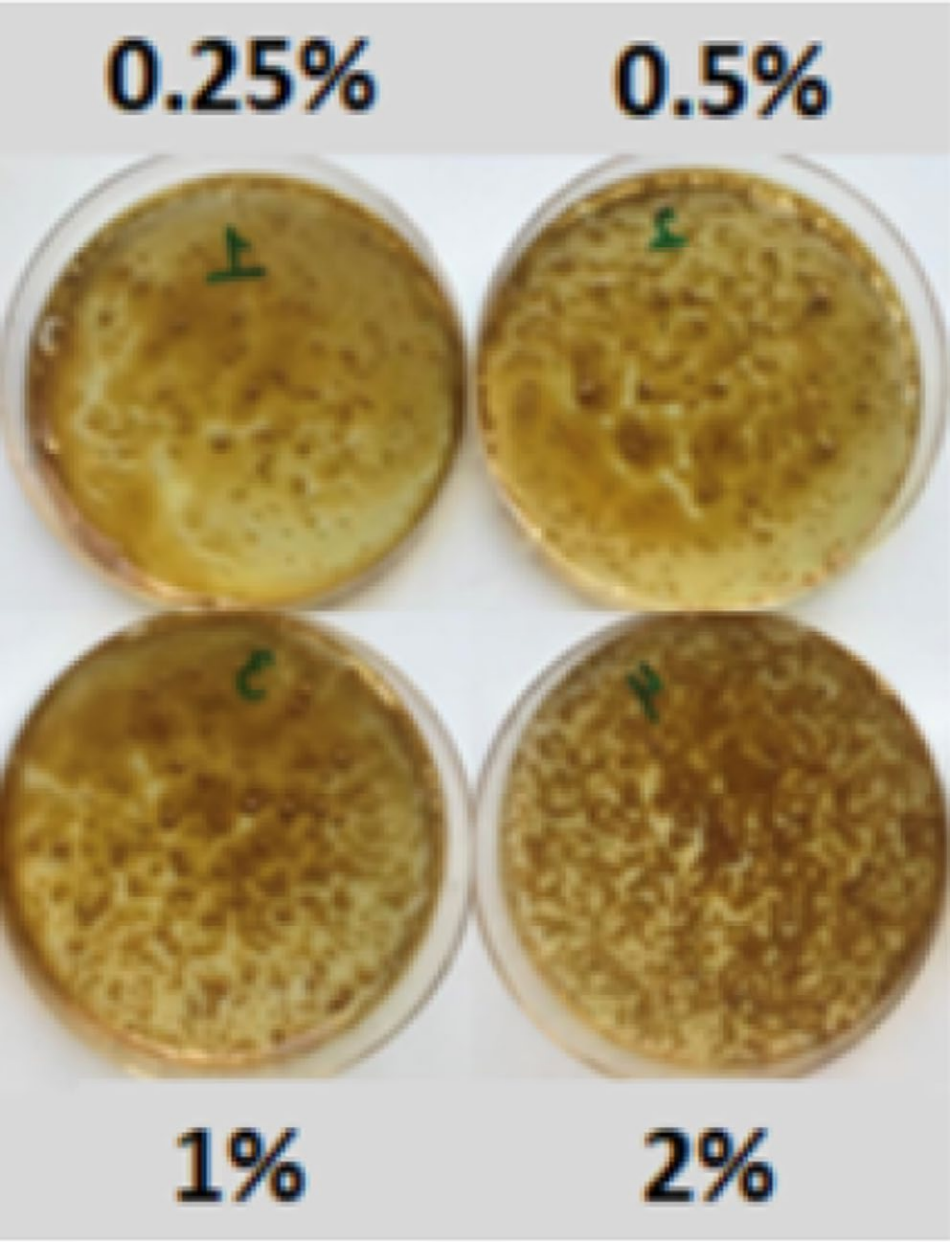
Visual images of mucoadhesive oral films containing 1% OLE by w/v and different ratios of TEO. Photograph courtesy of “Kubra Goktas.” Copyright 2023.

Figure 9 demonstrates the visual appearances of the films. It was observed that the films lose their mucoadhesion nature as well as homogeneity as the microcapsule ratio increases. Therefore, it has been determined that the concentration of microcapsules should not exceed 250 mg/100 mL film solution.

### 3.7 Further Characterization of Mucoadhesive Film Containing OLE and TEO Microcapsules

A series of mechanical and physical characterization tests were performed for the resulting optimized mucoadhesive film. As a result of tensile strength test (Figure S10), the maximum applied force on mucoadhesive oral film was found to be 0.02105 kN. After this point, the film structure started to break down, and the maximum strain of the film was determined as 12.38%. The abrasion and swelling capacity of the film were also determined, for which the percentage changes over time are provided in Figure S11. The mucoadhesive oral film has demonstrated a swelling capacity of up to 250 times itself. Nevertheless, the pH of solution and film were compatible with the oral mucosa structure. The average disintegration time was determined as 58.33 s. According to the weight and thickness measurements, the film structure was found to be homogeneous with a low standard deviation. The mucoadhesive film also displayed a desirable flexibility with a folding endurance of over 100 times. For ease, these results were summarized in Table 5.

**TABLE 5:** Summary of characterization results obtained for the optimized mucoadhesive oral film.

Viscosity (Pa s) ± SD	Solution pH ± SD	FILM pH ± SD	Disintegration time (s) ± SD	Thickness (mm) ± SD	Weight (mg) ± SD	Folding endurance	Mucoadhesion force (kN) ± SD	Contact angle ± SD
0.09 ± 0.01	7.49 ± 0.2	7.42 ± 0.1	58.33 ± 0.2	0.23 ± 0.01	61.98 ± 0.1	> 100	0.39 ± 0.02	31 ± 0.2

**FIGURE 10:**
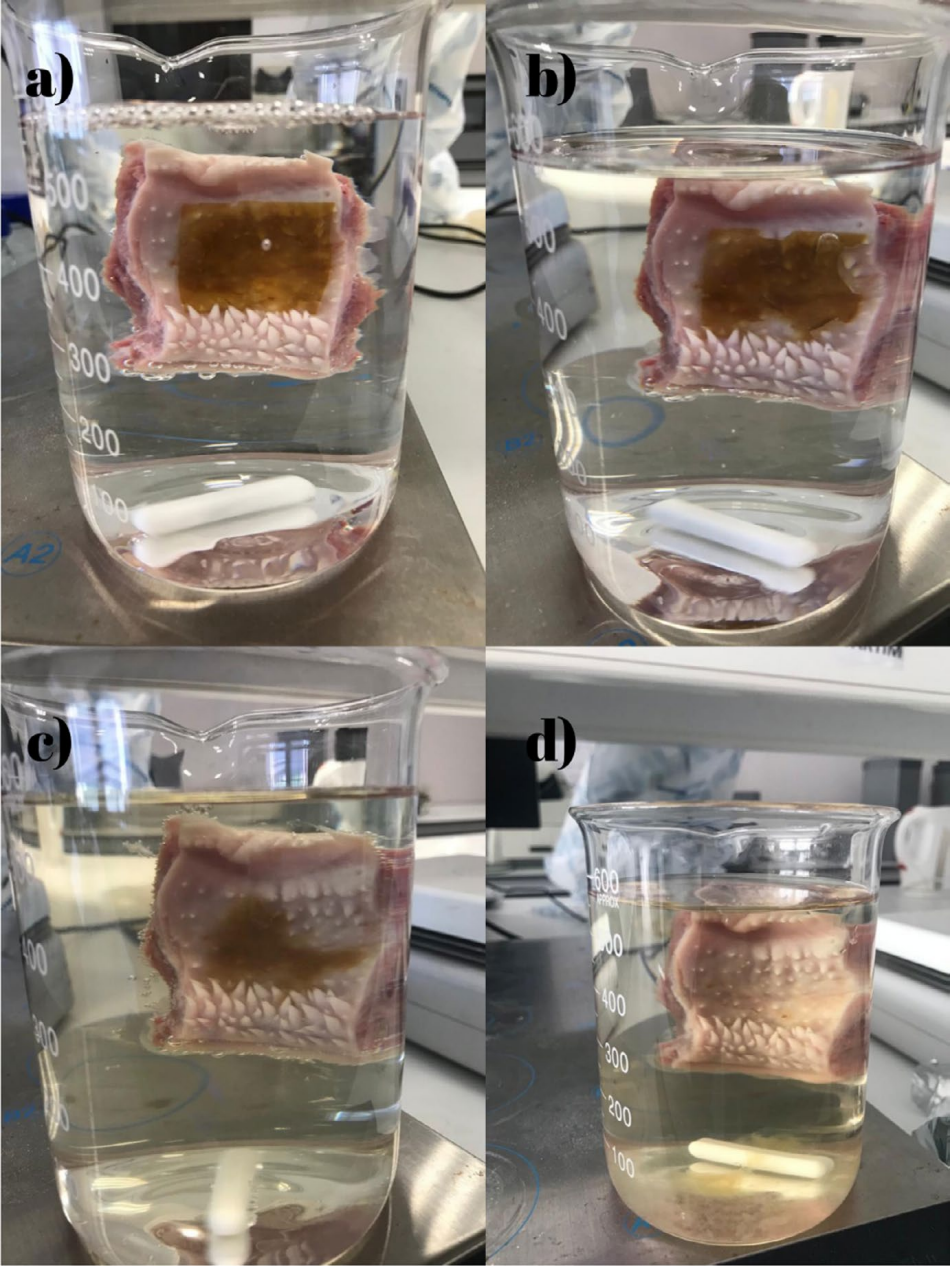
Mucoadhesion time test (a) initial state, *t* = 0, (b) after 4 h, (c) after 12 h, and (d) after 24 h. Photograph courtesy of “Kubra Goktas.” Copyright 2023.

Furthermore, using a cow buccal mucosa, ex vivo mucoadhesion time was measured for the final optimized films. Figure 10 shows the images taken during the test at different time intervals over a 1‐day period. Throughout the test, the film maintained its stability and adhesion ability until it was fully dissolved in the mucosal structure.

### 3.8 Antioxidant Capacity of the Film

The antioxidant capacity of the final optimized film formulation was determined as 0.15 mM TEAC/g (TEAC: Trolox‐equivalent antioxidant capacity). It was observed that this antioxidant capacity arose primarily from the OLE content and also from TEO, to lesser extent, as the control film containing no TEO microcapsules or OLE did not exhibit any antioxidant activity. According to the analysis, the antioxidant capacity of crude olive leaf extract and TEO were found to be 25.58 mM and 6.83 mM TEAC/g, respectively.

### 3.9 Cytotoxicity of the Film

**FIGURE 11:**
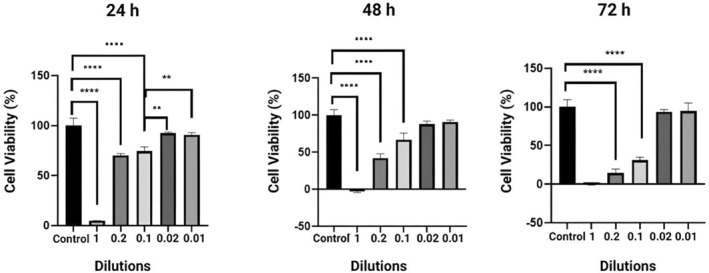
Results of Alamar Blue cell viability test performed after 24, 48, and 72 h of the optimized film by varying the dose through serial dilutions. The * symbol in the graphs indicates statistical significance; * was measured as *p* < 0.05, **p < 0.01, ***p < 0.001, and ****p < 0.0001.

The cytotoxic effects of the optimized film were analyzed in comparison with crude olive leaf extract, TEO, and a control film with no natural compounds present. This analysis aimed to determine the level of cytotoxicity of the film and identify the source of this effect if any, similar to the analysis performed for antioxidant capacity. The calculated cell viability (%) of the resulting film is represented in Figure 11. From Figure 11, it is clear that the final optimized film without dilution significantly reduced cell viability in the first 24 h, and it was found that this cytotoxic effect arose from both OLE and carvacrol component of TEO when used as they are, without any dilutions (24‐h cell viability results obtained for OLE, thymol and carvacrol standards are provided in Figure S12).

However, cell viability rose to more than 50% when the film extract was diluted to a 1/5 ratio, and kept increasing with increased dilutions. In comparison to the control film, there was no discernible change at dilutions of 1/50 and 1/100, meaning that mucoadhesive film extract at dilutions of equal to or greater 1/50 is non‐toxic to L929 cells for the first 24 h and remained the same for a total of 3 days.

#### 3.9.1 Antiviral, Antibacterial, and Antifungal Analysis

As mentioned previously, in this study, only a preliminary antimicrobial analysis was performed for the resulting film by an external accredited laboratory. Results obtained from these antimicrobial tests were summarized in Table 6 for convenience. All the original analysis reports are also provided in Appendix S1.

**TABLE 6:** Summary of preliminary antimicrobial analysis results.

Type of analysis	Type of microorganism	Result^a^	Contact time (min)
Bacterial	*Escherichia coli*	< 5 Log	1
	*Staphylococcus aureus*	> 5 Log (significant reduction)	
	*Pseudomonas aureginosa*	< 5 Log	
	*Enterococcus hirae*	< 5 Log	
Fungicidal	*Candida albicans*	No reduction	15
	*Aspergillus brasiliensis*	No reduction	
Virucidal	*Poliovirus Type 1*	0.17 Log (32.39% viral reduction)	2
	*Murine Novovirus*	0.34 Log (54.29% viral reduction)	

^a^ Results were reported by an external laboratory according to the standard method of analysis (TS EN 14476 & TS EN 13727, which are equivalent to ISO 21702 & ISO 22196, respectively). Source of strains: *E. coli* ATCC 10538; *S. aureus* ATCC 6538; *P. aeruginosa* ATCC 15442; *E. hirae* ATCC 10541; *C. albicans* ATCC 10231; *A. brasiliensis* ATCC 16404; *Poliovirus Type 1* Sabin strain_LSc‐2ab; *M. norovirus* S99 Berlin Strain ATCC VR‐1508.

According to the preliminary results of antimicrobial tests, the resulting film did not show a remarkable reduction against *E. coli* , *P. aureginosa*, and *E. hirae* bacteria in 1 min; however, it showed the desired logarithmic reduction against *S. aureus* bacteria and was reported to be effective. It was evaluated that the mucoadhesive oral film was fungicidal ineffective against *A. brasiliensis* and *C. albicans* species in 15 min. From the virucidal analysis, a viral reduction of 32.39% against *Poliovirus Type 1* and 54.29% against *M. Norovirus* was observed within 2 min. The demonstrated potential antibacterial and antiviral effects of the developed mucoadhesive film were expected to have risen from both the olive leaf extract and thyme essential oil contents (Ghaderi Ghahfarokhi et al. 2016; Radünz et al. 2020; Marchese et al. 2016). It is also known that these effects may increase with the increase of the contact time under more controlled experimental conditions.

## 4 Conclusion

In this investigation, a novel dual‐carrier system, integrating encapsulation and mucoadhesive film technology, was developed through optimization studies performed based on Box–Behnken experimental design. The TEO content and polymer composition of the optimized mucoadhesive film were determined successfully. Comprehensive physicochemical and mechanical characterization tests showed that the resulting mucoadhesive film had good tensile strength, swelling capacity, flexibility, and excellent compatibility with oral mucosa. Notably, strong adhesion to cow cheek mucosa was demonstrated. Although antimicrobial evaluations revealed no significant reductions according to the standard antibacterial, antifungal, and antiviral analysis conditions, the preliminary results confirmed that the optimized film exhibited potential antimicrobial activity. This suggests that further investigation has to be performed to determine the effects of film composition, concentration, and the contact time of analysis on the antimicrobial activity and hence, to improve the effectiveness of mucoadhesive film formulations for their potential use in drug delivery systems.

## Author Contributions

**Kubra Goktas:** data curation (equal), investigation (equal), software (equal), writing – original draft (equal). **Dilek Yalcin:** conceptualization (equal), data curation (equal), investigation (equal), methodology (equal), supervision (equal), writing – review and editing (equal). **Cansu Erdem:** data curation (equal), investigation (equal), writing – original draft (equal). **Beyza Tutku Bicakci:** data curation (equal), investigation (equal), writing – original draft (equal). **Oguz Bayraktar:** conceptualization (equal), funding acquisition (lead), investigation (equal), methodology (equal), supervision (equal), writing – review and editing (equal).

## Conflicts of Interest

The authors declare no conflicts of interest.

## Supporting information


Appendix S1

## Data Availability

The data that support the findings of this study are available on request from the corresponding authors.
