# Supplementary material for: Optimization of Mucoadhesive Oral Films Containing Olive Leaf Extract and Microencapsulated Thyme Essential Oil With Potential Antimicrobial Activity
Source: Food Sci Nutr. 2025 Jan 31;13(2):e4603. doi: 10.1002/fsn3.4603 (PMC11782971; doi:10.1002/fsn3.4603)
Supplement: Supplementary file 1 — Appendix S1 [file FSN3-13-e4603-s001.docx]

**Optimization of mucoadhesive oral films containing olive leaf extract and microencapsulated thyme essential oil with potential antimicrobial activity**

Kubra Goktas^1^, Dilek Yalcin^1*^, Cansu Erdem^1^, Beyza Tutku Bicakci^1^, Oguz Bayraktar^1*^

^1^ Department of Bioengineering, Ege University, Bornova, 35040, Izmir, Turkey

****Corresponding Author(s)***

**Dilek Yalcin**

Email: [dilek.yalcin@mail.ege.edu.tr](mailto:dilek.yalcin@mail.ege.edu.tr)

**Oguz Bayraktar**

Email: [oguz.bayraktar@ege.edu.tr](mailto:oguz.bayraktar@ege.edu.tr)

**SUPPORTING INFORMATION**

Characterization of olive leaf extract and thyme essential oil, experimental design tables for TEO encapsulation and film formulations, ANOVA results and response-surface plots for average microcapsule diameter and encapsulation yield, characterization results for the optimized mucoadhesive film and antimicrobial test results.

**Supporting Information**

**Characterization of Olive Leaf Extract**

***Determination of Total Antioxidant Capacity of Olive Leaf Extract (OLE)***

Total antioxidant capacity was determined by the ABTS method as Trolox equivalent using UV spectrophotometer (Synergy™ HTX Multi-Mode Microplate Reader). The 7mM ammonium ABTS salt was dissolved in water and activated by stirring with 2.45 mM K_2_S_8_O_2_ in the dark at 12-16 hrs. The prepared ABTS solution was diluted with methanol and its absorbance was adjusted to be 0.7 at 734 nm. 10 μL of the sample was mixed with 200 μL of ABTS solution in 3 repetitions in 96 Plate wells and absorbance was measured after 30 minutes. The total radical scavenging capacity was calculated with Trolox's absorbance reduction, and the result was expressed in terms of Trolox equivalent antioxidant capacity per gram of sample (mM TEAC/g) [1]. In this study, the antioxidant capacity of OLE was determined as 25.58 mM TEAC/g. In another study, the antioxidant capacity was reported as 11.62 mM TEAC/g [2].

***Determination of Total Phenolic Content of Olive Leaf Extract***

The total phenolic content of the extract was determined through the Folin-Ciocalteu colorimetric method, using gallic acid as the standard [3]. 3 replicates of 20 μl samples were inoculated into 96 Plate wells. 20 μl methanol was added to the control group. In the dark environment, 100 μl of Folin-Ciocalteu reagent was added to the samples and after 5 minutes, 80 μl of a saturated sodium carbonate solution was added. Absorbance at 725 nm was measured after 1-hour incubation in the dark at room temperature. The total phenolic content is expressed in milligrams of gallic acid equivalents (GAE) per gram of dry material [4]. The total amount of phenolic substances was determined as 222,26 mg GAE / g while it was reported as 197.42 mg GAE/g by others [5].

***Determination of Oleuropein in Olive Leaf Extract***

The quantity of oleuropein in the extract was determined by HPLC analysis (Thermo Scientific Ultimate 3000), for which 200 mL of water, 200 mL of acetonitrile, and 1 mL of acetic acid were combined to make the mobile phase. For analysis, 0.1 g of OLE dissolved in 10 ml of ethanol and measurements were taken at a wavelength of 280 nm. Figure S1 shows the corresponding chromatogram obtained for OLE. According to this, the amount of oleuropein was determined as 309 mg in 1 g of olive leaf extract. Notwithstanding, Altiok and co-workers reported the amount of oleuropein in 1 g of olive leaf extract as 134.4 mg in their study [6].


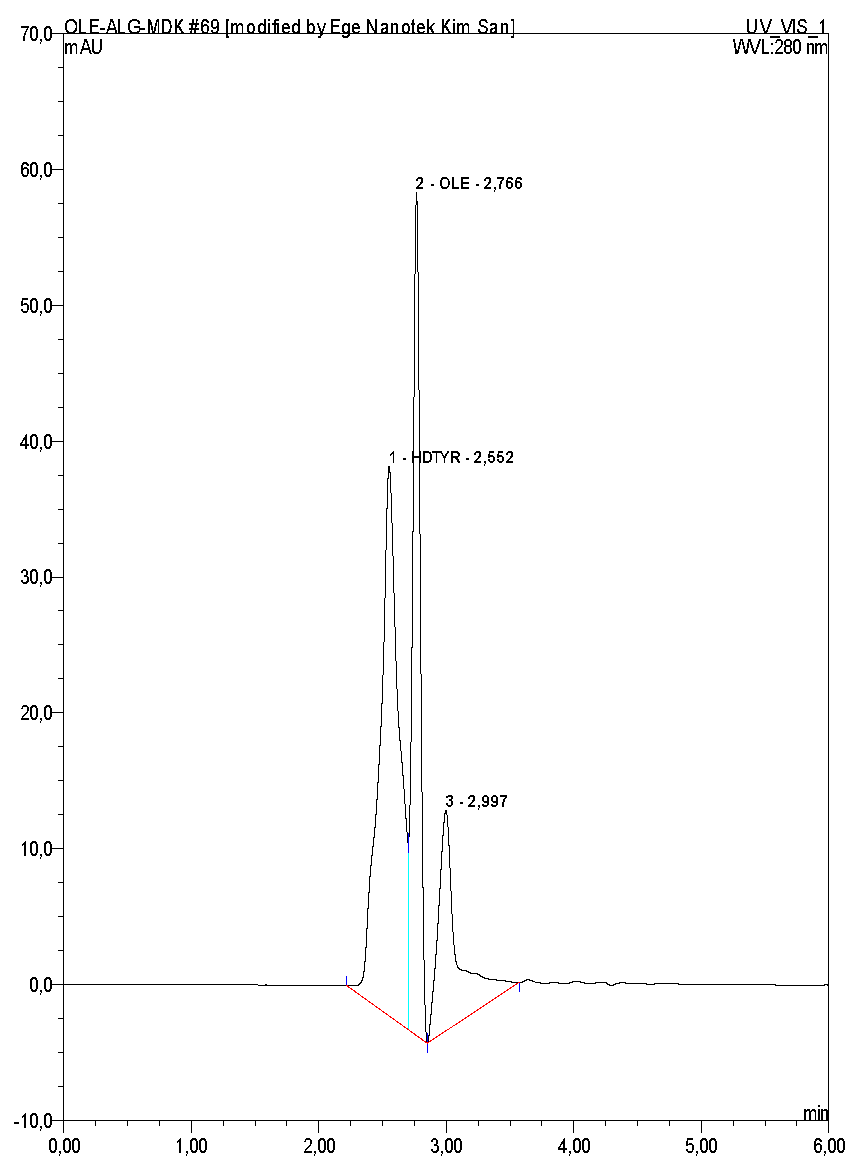


**Figure S1.** HPLC chromatogram of olive leaf extract.

**FTIR Analysis of Olive Leaf Extract**

FTIR analysis was carried out to determine the functional groups of the powdered olive leaf extract. Figure S2 shows the characteristic peaks of OLE in the FTIR spectrum. The broad peak seen in the range of 3200-3300 cm^-1^ refers to N-H and O-H bonds. The peak at 2929 cm^-1^ shows the C-H vibration of the –CH_3_ and –CH_2_ functional groups. The C=O carboxylic acid group, and C-O and O-H groups of olive leaf extract are reflected by the peaks at 1694 cm^-1^ and 1613 cm^-1^, respectively. The small peak seen at 1513 cm^-1^ indicates N-H vibration while peak at 1445 cm^-1^ shows the vibration of methylene groups. The sharp and strong peak at 1070 cm^-1^ is interpreted as the C-N stretching vibrations of aliphatic amines [7].


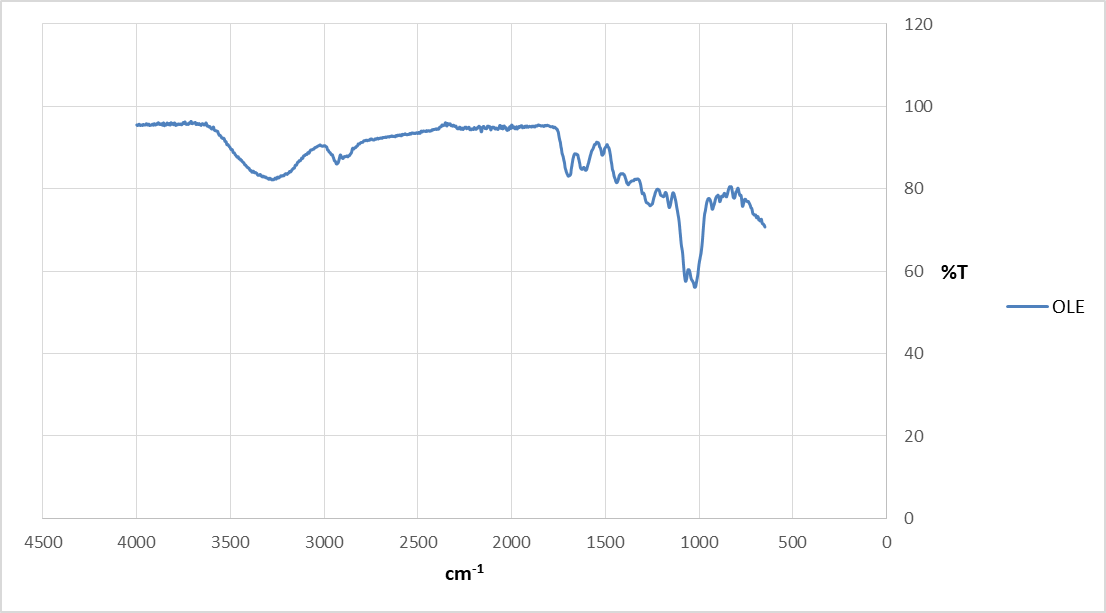


**Figure S2.** FTIR spectrum of olive leaf extract.

**Characterization of Thyme Essential Oil**

Locally obtained TEO was first characterized through GC-7890 B with HP-INNOWAX capillary column (0.25 μm film, 0.320 mm diameter, and 60 m length) coupled to a mass spectrometer series MSD 5977 A (Agilent 6550 iFunnel high resolution Accurate Mass Q-TOF/MS). The flow rate of the carrier helium gas was 1 mL/min. A temperature gradient was applied during analysis, where the program started at 50 °C temperature, was held at 240 °C for 2 min (heating rate=4 °C /min), and then temperature was increased to 260 °C with 10 °C/min of heating rate and maintained for 5 min. Interface temperature for GC-MS was 250 °C, and the injection was performed in the split mode (1:50), where the injection volume was 2.0 μL. The electron impact energy was set at 70 eV, and data were collected in the range of 50–500 atomic mass units. Compound identification was performed based on peak-matching with Wiley’s MS spectra database while the integrations were done using a built-in software called MassHunter.


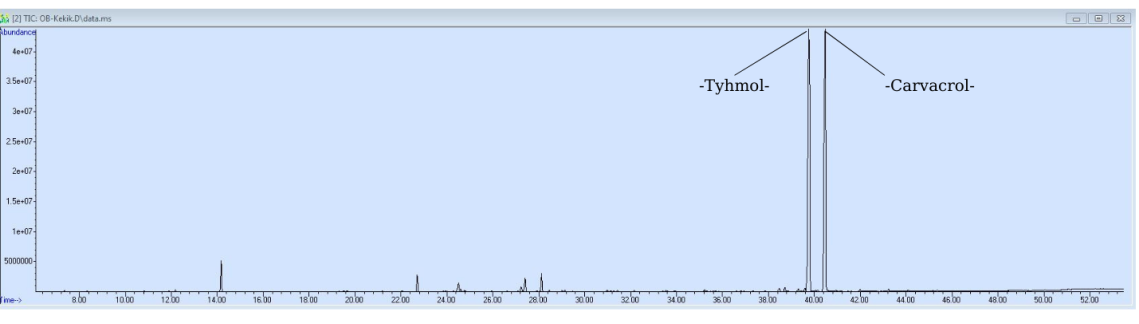

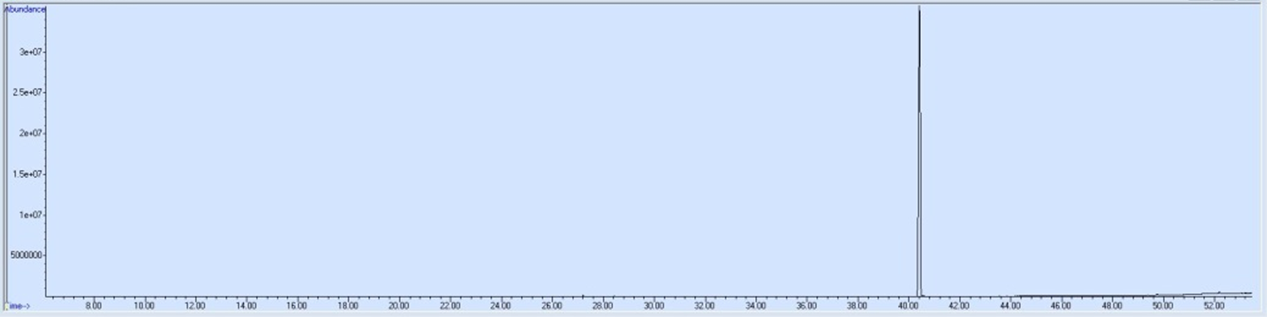

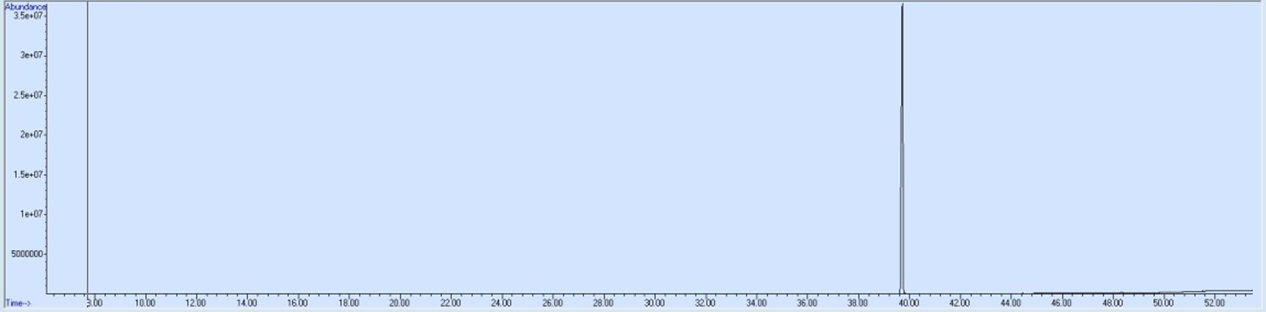


**Figure S3.** GC chromatograms of A. Carvacrol Standard, B. Thymol Standard,

and C. Thyme Essential Oil.

**Table S1.** Composition of thyme essential oil according to the GC-MS analysis

| **PK** | **RT** | **Area Pct** | **Library/ID** |
| --- | --- | --- | --- |
| 1 | 7.3514 | 0.1151 | ALPHA-PINENE |
| 2 | 8.3422 | 0.0636 | Camphene |
| 3 | 10.82 | 0.0466 | Myrcene |
| 4 | 11.907 | 0.0763 | l-Limonene |
| 5 | 12.1867 | 0.1721 | 1,8-Cineole |
| 6 | 14.1732 | 2,6828 | Benzene, 1-methyl-3-(1-methylethyl)- |
| 7 | 19.2987 | 0,0454 | Ethanone, 1-(1,3-dimethyl-3-cyclohexen-1-yl)- |
| 8 | 19.492 | 0.1121 | Benzene, 1-methyl-2-(2-propenyl)- (CAS) |
| 9 | 19.6586 | 0.0573 | 1 OCTEN 3 OL |
| 10 | 20.4552 | 0.0414 | LINALOOL OXIDE CIS EPOXYLINALOOL |
| 11 | 21.193 | 0.0579 | Copaene |
| 12 | 22.0569 | 0.0872 | Camphor |
| 13 | 22.7111 | 1.4553 | LINALOOL L |
| 14 | 23.8347 | 0.0542 | Bornyl formate |
| 15 | 23.921 | 0.0476 | Acetic acid, 1,7,7-trimethyl-bicyclo[2.2.1]hept-2-yl ester |
| 16 | 24.2776 | 0.0821 | Benzene, 2-methoxy-4-methyl-1-(1-methylethyl)- |
| 17 | 24.5037 | 1.1306 | 3-Cyclohexen-1-ol, 4-methyl-1-(1-methylethyl)- (CAS) 4-Terpineol |
| 18 | 24.6016 | 0.2314 | CARVACROL METHYL ETHER |
| 19 | 24.7602 | 0.1221 | Alloaromadendrene |
| 20 | 25.9434 | 0.0728 | Pulegone |
| 21 | 26.6076 | 0.0861 | Humulene |
| 22 | 27.094 | 0.0766 | ALPHA. AMORPHENE |
| 23 | 27.2342 | 0.4848 | .ALPHA. TERPINEOL |
| 24 | 27.406 | 1.3901 | BORNEOL L |
| 25 | 27.9355 | 0.0991 | 2-Cyclohexen-1-one, 6-methyl-3(1-methylethyl)- (Carvenone) |
| 26 | 28.1262 | 1.7023 | Bisabolene |
| 27 | 28.4571 | 0.1541 | 2-Cyclohexen-1-one, 2-methyl-5-(1-methylethenyl)-, (S)- |
| 28 | 29.0223 | 0.094 | Cadinene |
| 29 | 29.1247 | 0.1452 | Naphthalene, 1,2,3,4,4a,5,6,8a-octahydro-7-methyl-4-methylene-1-(1-methylethyl)-, (1.alpha.,4a.beta.,8a.alpha.)- |
| 30 | 29.4564 | 0.0405 | Benzene, 1-(1,5-dimethyl-4-hexenyl)-4-methyl- |
| 31 | 30.9809 | 0.1733 | Benzene, 1-methoxy-4-(2-propenyl)- (CAS) p-Allylanisole Anisole, p-allyl- Methyl chavicol |
| 32 | 31.1331 | 0.0897 | 1S,CIS-CALAMENENE |
| 33 | 31.2856 | 0.052 | trans-Geraniol, Guaniol, Lemonol, Geraniol, Geranyl alcohol |
| 34 | 31.4307 | 0.1146 | PARA-CYMEN-8-OL |
| 35 | 32.1721 | 0.1254 | CARVACRYL ACETATE |
| 36 | 33.4013 | 0.0572 | 1-Thienylcyclohexene |
| 37 | 33.5409 | 0.1303 | 1(2H)-Naphthalenone, 3,4,5,6,7,8-hexahydro-7-methyl- (CAS) |
| 38 | 33.6196 | 0.0708 | 2-Cyclohexen-1-one, 3-methyl-6-(1-methylethylidene)- (CAS) Piperitenone |
| 39 | 33.9405 | 0.111 | 2,4-Hexadiene, 2,5-dimethyl-, Biisobutenyl, Biisocrotyl, Diisocrotyl, 2,5-Dimethyl-2,4-hexadiene |
| 40 | 35.2185 | 0.2245 | Caryophyllene oxide |
| 41 | 35.3357 | 0.0536 | (3E)-1-cyclopentylidene-3-methyl-3-penten-2-one 3-Penten-2-one, 1-cyclopentylidene-3-methyl-, (E)- (CAS) |
| 42 | 35.6935 | 0.0564 | Benzene, 1,2-dimethoxy-4-(2-propenyl)- (CAS) Methyleugenol Methyl Eugenol 1- |
| 43 | 36.6183 | 0.0361 | 12-Oxabicyclo[9.1.0]dodeca-3,7-diene, 1,5,5,8-tetramethyl-, [1R-(1R@,3E,7E,11R@)]- |
| 44 | 36.8068 | 0.0778 | .alpha.-Caryophyllene alcohol 4,8-Methanoazulen-9-ol, decahydro-2,2,4,8-tetramethyl-, stereoisomer (CAS) |
| 45 | 36.9339 | 0.0481 | Naphthalene, 1,2,3,4,4a,7-hexahydro-1,6-dimethyl-4-(1-methylethyl)- |
| 46 | 37.3404 | 0.0676 | (-)-Globulol |
| 47 | 37.874 | 0.0676 | Benzenemethanol, 4-(1-methylethyl)- |
| 48 | 38.4725 | 0.3529 | (+) spathulenol |
| 49 | 38.7168 | 0.3858 | D,L-p-Fluorophenylalanine |
| 50 | 39.3128 | 0.2914 | Phenol, 5-methyl-2-(1-methylethyl)- (CAS) Thymol m-Thymol p-Cymen-3-ol Thyme camphor |
| 51 | 39.4362 | 0.0644 | 3-Allyl-6-methoxyphenol |
| 52 | 39.5929 | 0.3671 | t-Cadinol Cedrelanol |
| **53** | **39.7732** | **42.5571** | **Thymol (C_10_H_14_O, MW: 150.22 g/mol)** |
| 54 | 40.0613 | 0.1325 | Clovene |
| 55 | 40.1489 | 0.1596 | p-Cymen-2-ol Antioxine Isothymol |
| **56** | **40.4736** | **41.5283** | **Carvacrol (C_10_H_14_O, MW: 150.22 g/mol)** |
| 57 | 40.8355 | 0.0533 | 1H-3a,7-Methanoazulene, 2,3,6,7,8,8a-hexahydro-1,4,9,9-tetramethyl-, (1.alpha.,3a.alpha.,7.alpha.,8a.beta.)- |
| 58 | 40.9316 | 0.1448 | Naphthalene, 1,6-dimethyl-4-(1-methylethyl)- |
| 59 | 41.1491 | 0.0555 | P-CYMEN-.ALPHA.-OL 1-HYDROXYMETHYL-4-ISOPROPYLBENZENE Cuminol p-Cymen-7-ol |
| 60 | 41.9998 | 0.2056 | Benzene, 2-methoxy-1,3,4-trimethyl- Anisole, 2,3,6-trimethyl- |
| 61 | 42.1422 | 0.0493 | 3-Cyclohexen-1-carboxaldehyde, 3,4-dimethyl- |
| 62 | 42.4129 | 0.0735 | caryophylla-4(12),8(13)-dien-5.beta.-ol |
| 63 | 43.0258 | 0.05 | 2-Methylene-3-(cis-prop-1-en-1-yl)cyclohexanone Cyclohexanone, 2-methylene-3-(1-propenyl)-, (Z)- (CAS) |
| 64 | 43.229 | 0.2406 | Isoaromadendrene epoxide |
| 65 | 43.3047 | 0.046 | Benzene, 1,3-dimethyl-5-(1-methylethyl)- Cumene, 3,5-dimethyl- 1-Isopropyl-3,5-dimethylbenzene |
| 66 | 43.613 | 0.0703 | 1 Labd-14-ene, 8,13-epoxy-, (13R)- Manoyl oxide 8.alpha.,13-Epoxylabd-14-ene |
| 67 | 43.7024 | 0.0519 | 1,4,7,10,13,16-Hexaoxacyclooctadecane Ethylene oxide cyclic hexamer |
| 68 | 44.0897 | 0.1613 | Caryophyllenol II |
| 69 | 44.9957 | 0.0507 | Tricyclo[7.2.0.0(2,6)]undecan-5-ol, 2,6,10,10-tetramethyl- (isomer 2) |
| 70 | 45.1675 | 0.0692 | (+)-6-OXOCAMPHENE |
| 71 | 45.3546 | 0.1159 | Benzene, 2-(butenyl)-5-(1,1-dimethylethyl)-1,3-dimethyl- |
| 72 | 45.5937 | 0.0377 | 21-KRONE-7 |
| 73 | 48.5386 | 0.02 | 1,4,7,10,13,16-Hexaoxacyclooctadecane (CAS) |
| 74 | 50.0322 | 0.0639 | 3-(4-Nitrophenyl)propiolic acid |
| 75 | 50.7786 | 0.0053 | 1,4,7,10,13,16-Hexaoxacyclooctadecane (CAS) |
| 76 | 50.9378 | 0.0136 | 1,4,7,10,13,16-Hexaoxacyclooctadecane |


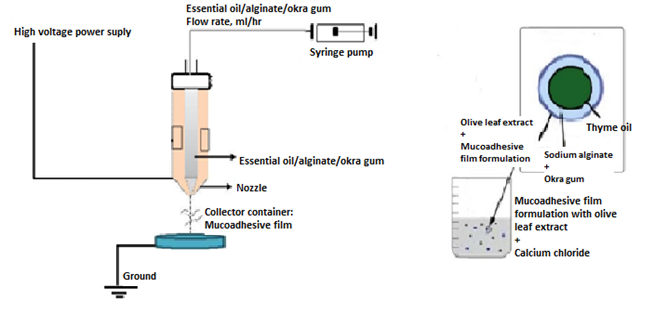


**Figure S4.** Laboratory-type electrospray system used for the encapsulation of thyme essential oil.

**Table S2.** Box-Behnken experimental design for thyme essential oil encapsulation

| **Experiment** | **A: Flow rate (ml/h)** | **B: Voltage (kV)** | **C: Distance (cm)** |
| --- | --- | --- | --- |
| 1 | 0.5 | 10 | 10 |
| 2 | 2.5 | 10 | 10 |
| 3 | 0.5 | 14 | 10 |
| 4 | 2.5 | 14 | 10 |
| 5 | 0.5 | 12 | 8 |
| 6 | 2.5 | 12 | 8 |
| 7 | 0.5 | 12 | 12 |
| 8 | 2.5 | 12 | 12 |
| 9 | 1.5 | 10 | 8 |
| 10 | 1.5 | 14 | 8 |
| 11 | 1.5 | 10 | 12 |
| 12 | 1.5 | 14 | 12 |
| 13 | 1.5 | 10 | 10 |
| 14 | 1.5 | 10 | 10 |
| 15 | 1.5 | 10 | 10 |

**Table S3.** Box-Behnken design for film formulation mixing ratios

| **Formulation number** | **Carbopol 934 (w/v)** | **HPMC (w/v)** | **Polyethylene glycol (w/v)** |
| --- | --- | --- | --- |
| F-1 | 0.1 | 1.5 | 0.4 |
| F-2 | 0.1 | 2 | 0.4 |
| F-3 | 0.1 | 2.5 | 0.4 |
| F-4 | 0.1 | 1.5 | 0.8 |
| F-5 | 0.1 | 2 | 0.8 |
| F-6 | 0.1 | 2.5 | 0.8 |
| F-7 | 0.1 | 1.5 | 1.2 |
| F-8 | 0.1 | 2 | 1.2 |
| F-9 | 0.1 | 2.5 | 1.2 |
| F-10 | 0.15 | 1.5 | 0.4 |
| F-11 | 0.15 | 2 | 0.4 |
| F-12 | 0.15 | 2.5 | 0.4 |
| F-13 | 0.15 | 1.5 | 0.8 |
| F-14 | 0.15 | 2 | 0.8 |
| F-15 | 0.15 | 2.5 | 0.8 |
| F-16 | 0.15 | 1.5 | 1.2 |
| F-17 | 0.15 | 2 | 1.2 |
| F-18 | 0.15 | 2.5 | 1.2 |
| F-19 | 0.2 | 1.5 | 0.4 |
| F-20 | 0.2 | 2 | 0.4 |
| F-21 | 0.2 | 2.5 | 0.4 |
| F-22 | 0.2 | 1.5 | 0.8 |
| F-23 | 0.2 | 2 | 0.8 |
| F-24 | 0.2 | 2.5 | 0.8 |
| F-25 | 0.2 | 1.5 | 1.2 |
| F-26 | 0.2 | 2 | 1.2 |
| F-27 | 0.2 | 2.5 | 1.2 |

**Table S4.** ANOVA Table For Average Microcapsule Diameter.

(R²: 0.9990 ; Adjusted R²: 0.9933)

| **Source** | **Sum of Squares** | **df** | **Mean Square** | **F-value** | **p-value** |  |
| --- | --- | --- | --- | --- | --- | --- |
| **Model** | 0.1018 | 12 | 0.0085 | 173.10 | 0.0058 | significant |
| A-Flow rate | 0.0018 | 1 | 0.0018 | 37.73 | 0.0255 |  |
| B-Potential difference | 0.0209 | 1 | 0.0209 | 426.13 | 0.0023 |  |
| C-Distance | 0.0008 | 1 | 0.0008 | 16.58 | 0.0554 |  |
| AB | 0.0001 | 1 | 0.0001 | 2.94 | 0.2286 |  |
| AC | 0.0003 | 1 | 0.0003 | 5.22 | 0.1496 |  |
| BC | 0.0044 | 1 | 0.0044 | 90.25 | 0.0109 |  |
| A² | 0.0376 | 1 | 0.0376 | 766.78 | 0.0013 |  |
| B² | 0.0003 | 1 | 0.0003 | 6.98 | 0.1184 |  |
| C² | 0.0035 | 1 | 0.0035 | 71.83 | 0.0136 |  |
| A²B | 0.0001 | 1 | 0.0001 | 1.59 | 0.3340 |  |
| A²C | 0.0013 | 1 | 0.0013 | 26.02 | 0.0363 |  |
| AB² | 0.0007 | 1 | 0.0007 | 14.73 | 0.0617 |  |
| **Pure Error** | 0.0001 | 2 | 0.0000 |  |  |  |
| **Cor Total** | 0.1019 | 14 |  |  |  |  |

**Table S5.** ANOVA Table For Encapsulation Yield

(R²: 0.9995 ; Adjusted R²: 0.9966)

| **Source** | **Sum of Squares** | **df** | **Mean Square** | **F-value** | **p-value** |  |
| --- | --- | --- | --- | --- | --- | --- |
| **Model** | 6598.38 | 12 | 549.86 | 341.48 | 0.0029 | significant |
| A-Flow rate | 688.01 | 1 | 688.01 | 427.27 | 0.0023 |  |
| B-Potential difference | 805.70 | 1 | 805.70 | 500.36 | 0.0019 |  |
| C-Distance | 760.93 | 1 | 760.93 | 472.56 | 0.0021 |  |
| AB | 134.90 | 1 | 134.90 | 83.781 | 0.0117 |  |
| AC | 243.36 | 1 | 243.36 | 151.13 | 0.0065 |  |
| BC | 919.60 | 1 | 919.60 | 571.10 | 0.0017 |  |
| A² | 1429.55 | 1 | 1429.55 | 887.79 | 0.0011 |  |
| B² | 5.85 | 1 | 5.85 | 3.63 | 0.1968 |  |
| C² | 156.80 | 1 | 156.80 | 97.37 | 0.0101 |  |
| A²B | 7.48 | 1 | 7.4884 | 4.6505 | 0.1637 |  |
| A²C | 412.27 | 1 | 412.27 | 256.03 | 0.0038 |  |
| AB² | 9.30 | 1 | 9.30 | 5.7815 | 0.1380 |  |
| **Pure Error** | 3.22 | 2 | 1.61 |  |  |  |
| **Cor Total** | 6601.60 | 14 |  |  |  |  |


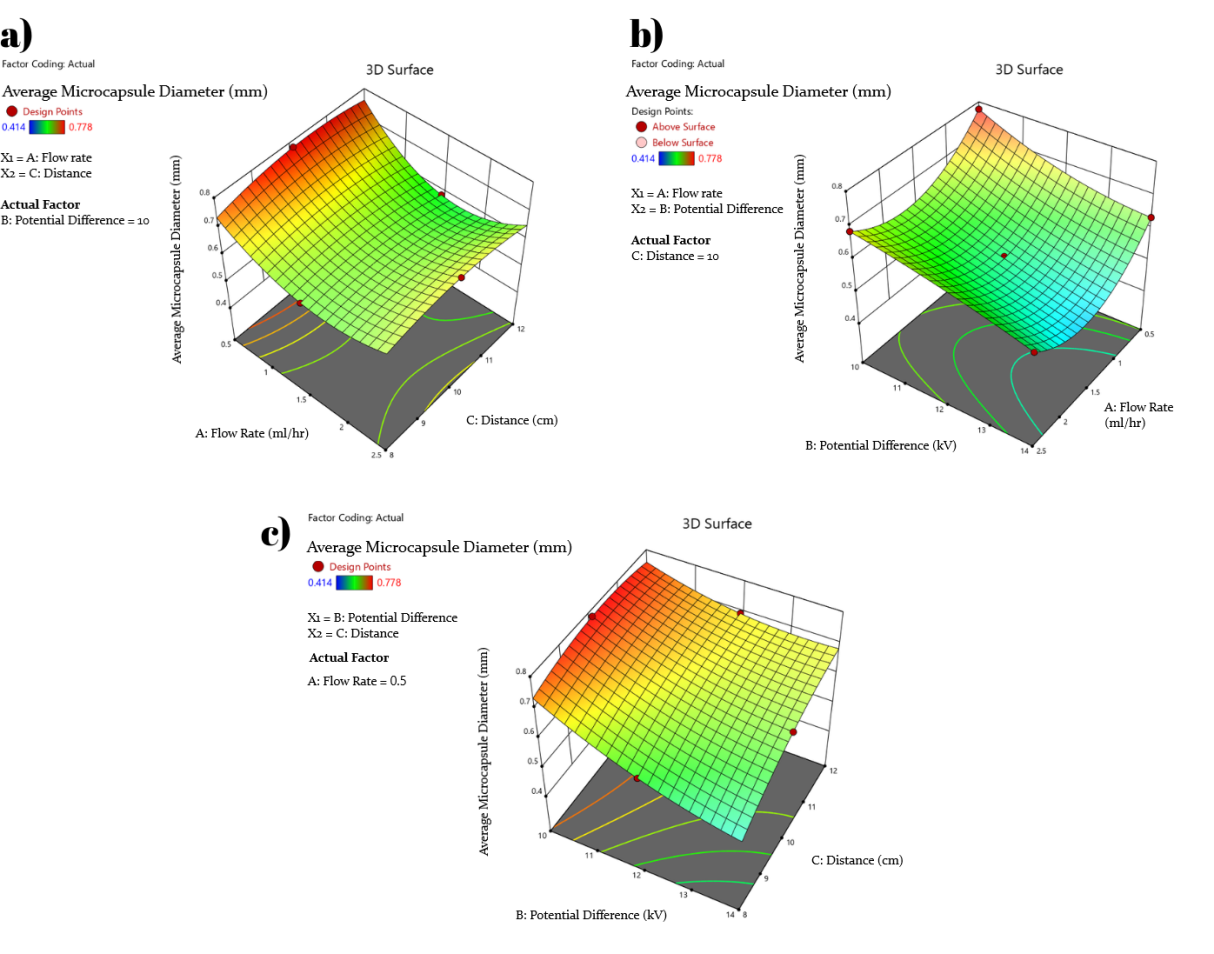


**Figure S5.** 3D Graphs for average microcapsule diameter changing with respect to a) Distance and Flow rate, b) Flow rate and Potential difference, and c) Potential difference and Distance.


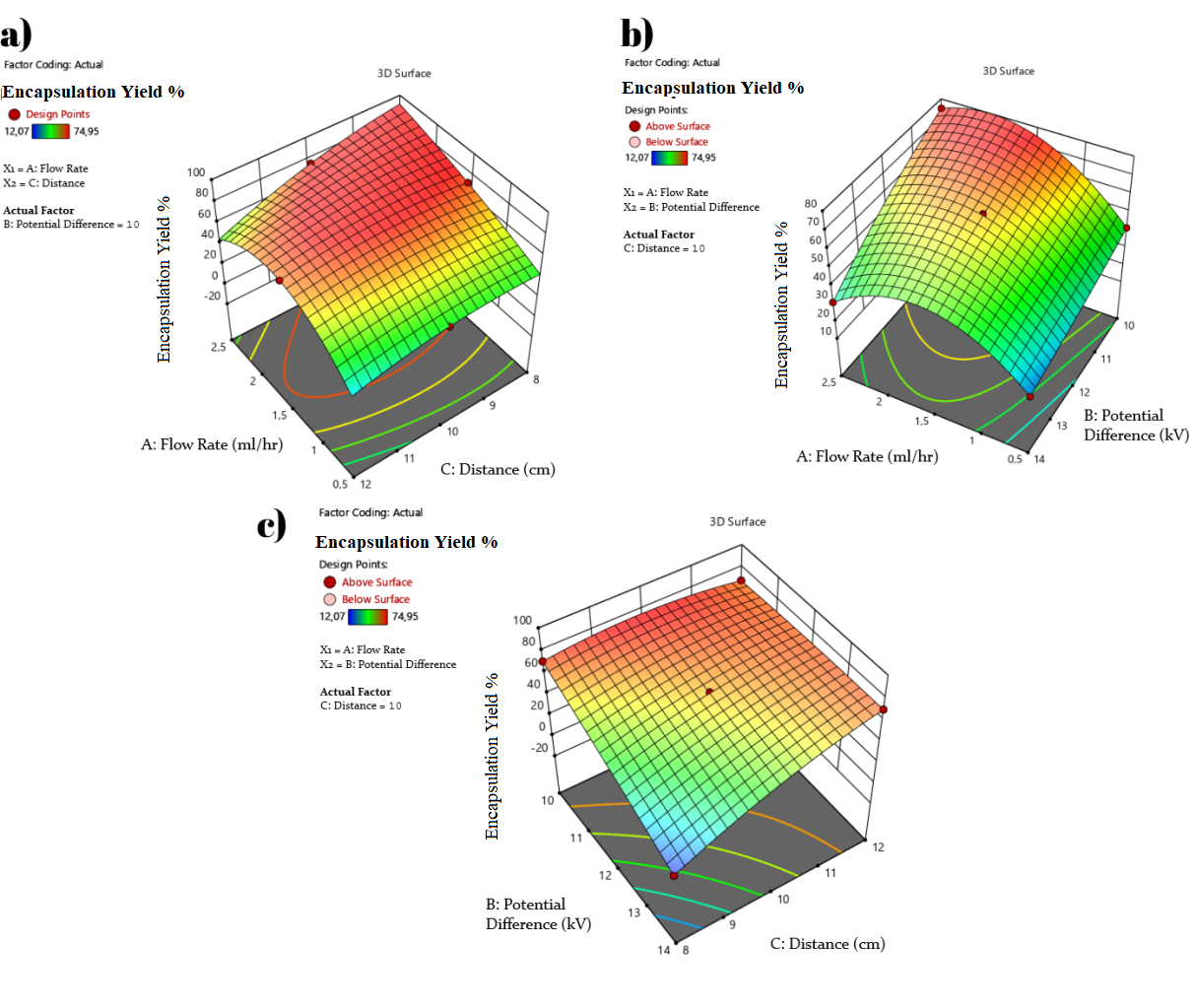


**Figure S6.** 3D Graphs for encapsulation yield changing with respect to a) Distance and Flow rate, b) Flow rate and Potential difference, and c) Potential difference and Distance.


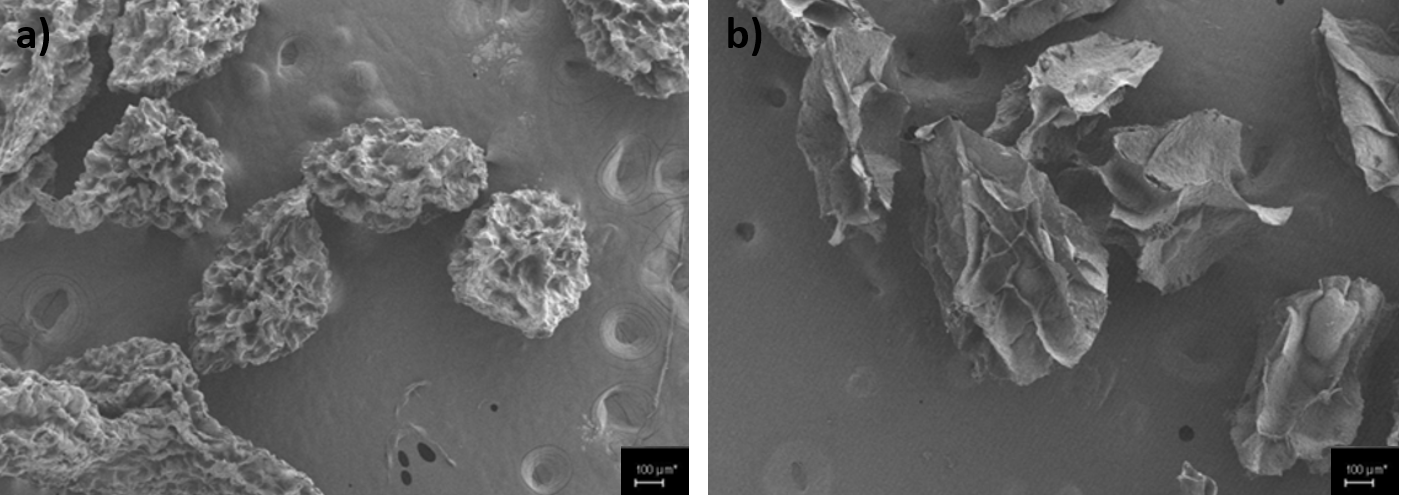


**Figure S7.** SEM images of microcapsules containing TEO a) before and b) after the release study. Scale bars are 100 µM. SEM images were taken at 100X magnification.

**
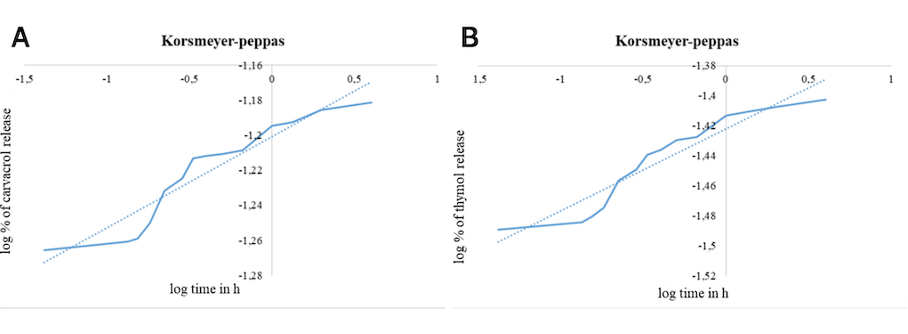
**

**Figure S8.** Release profiles obtained for A) carvacrol and B) thymol from the optimized microcapsules of TEO according to Korsmeyer-Peppas kinetic model

**Table S6.** Repeated weight and thickness measurements fort he selected mucoadhesive films

| **Formula number** | **C 934 (w/v)** | **HPMC (w/v)** | **PEG (w/v)** | **Thickness (mm) ± std** | **Weight (g) ± std** |
| --- | --- | --- | --- | --- | --- |
| **F-3** | 0.1 | 2.5 | 0.4 | 0.28 ± 0.17 | 0.15 ± 0.06 |
| **F-6** | 0.1 | 2.5 | 0.8 | 0.26 ± 0.13 | 0.06 ± 0.04 |
| **F-8** | 0.1 | 2 | 1.2 | 0.38 ± 0.05 | 0.07 ± 0.03 |
| **F-9** | 0.1 | 2.5 | 1.2 | 0.25 ± 0.08 | 0.06 ± 0.02 |
| **F-12** | 0.15 | 2.5 | 0.4 | 0.24 ± 0.09 | 0.05 ± 0.03 |


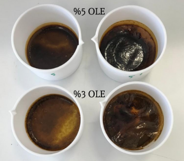


**Figure S9.** Mucoadhesive films made on the basis of Formulation F-8 with 5% and 3% OLE inclusion. Photograph courtesy of ‘Kubra Goktas’. Copyright 2023.


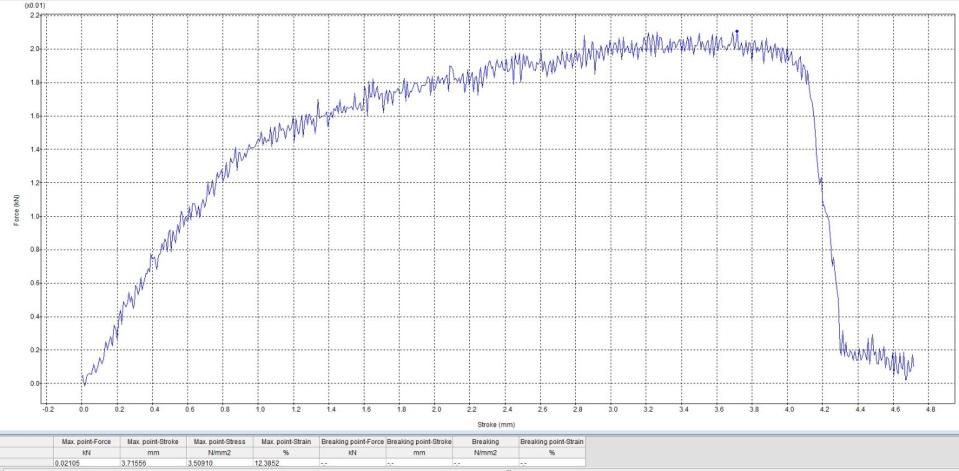


**Figure S10.** Force vs Strain plot for the optimized mucoadhesive film


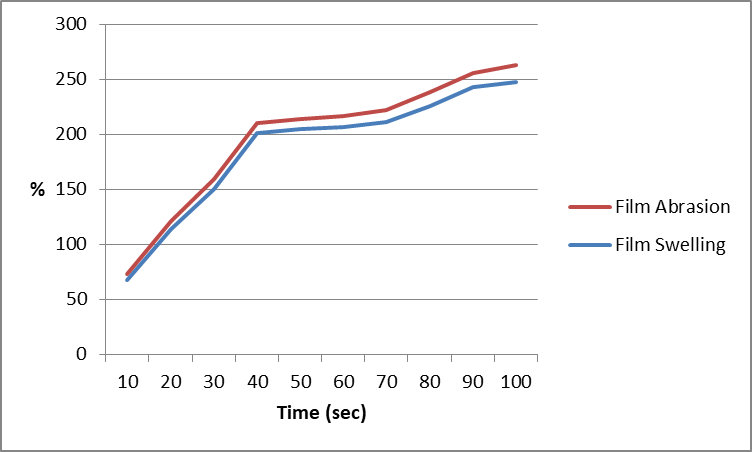


**Figure S11.** Average abrasion and swelling changes of mucoadhesive oral film.

**Cytotoxicity Analysis**


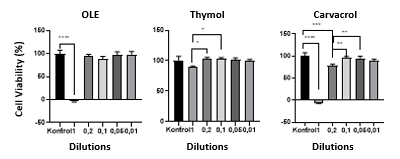


**Figure S12.** Results of Alamar Blue cell viability test performed for OLE, thymol and carvacrol standards after 24 hours by varying the dose through serial dilutions

***Antiviral, antibacterial and antifungal analysis***

**
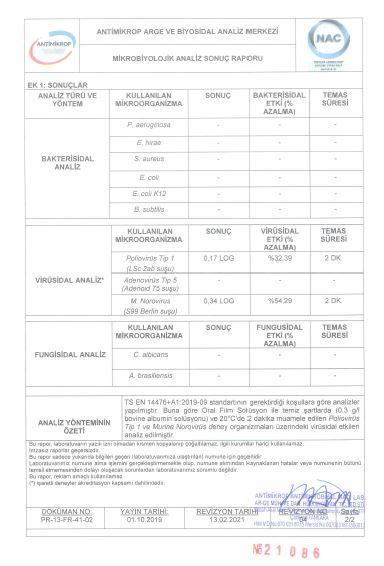
**

**
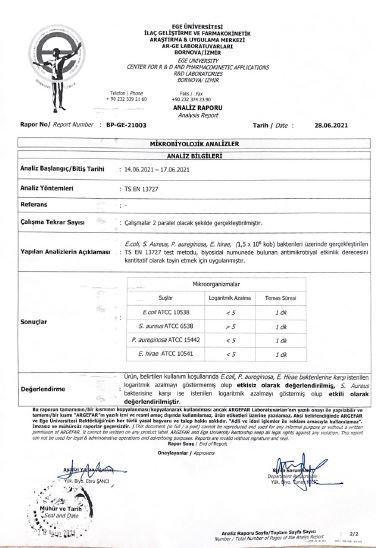
**

**REFERENCES**

1. Floegel A, Kim DO, Chung SJ, Koo SI, Chun OK. Comparison of ABTS/DPPH assays to measure antioxidant capacity in popular antioxidant-rich US foods. J Food Compos Anal. 2011;24:1043–8. Available from: http://dx.doi.org/10.1016/j.jfca.2011.01.008

2. Doğan G, Başal G, Bayraktar O, Ozyildiz F, Uzel A, Erdoğan I. Bioactive Sheath/Core nanofibers containing olive leaf extract. Microsc Res Tech. 2016;79:38–49.

3. Singleton VL, Rossi JAJ. Colorimetry to total phenolics with phosphomolybdic acid reagents. Am J Enol Vitic. 1965;16:144–58. Available from: http://garfield.library.upenn.edu/classics1985/A1985AUG6900001.pdf

4. Tawaha K, Alali FQ, Gharaibeh M, Mohammad M, El-Elimat T. Antioxidant activity and total phenolic content of selected Jordanian plant species. Food Chem. 2007;104:1372–8.

5. Aytul KK. Antimicrobial and antioxidant activities of olive leafextract and its food applications [Internet]. A Thesis Submitt. to Grad. Sch. Eng. Sci. İzmir Inst. Technol. Partial Fulfillment Requir. Degree master Sci. Biotechnol. 2010. Available from: http://library.iyte.edu.tr/tezler/master/biyoteknoloji/T000831.pdf

6. Altiok E, Bayçin D, Bayraktar O, Ülkü S. Isolation of polyphenols from the extracts of olive leaves (Olea europaea L.) by adsorption on silk fibroin. Sep Purif Technol. 2008;62:342–8.

7. M. Awwad A, M. Salem N, O. Abdeen A. Biosynthesis of Silver Nanoparticles using *Olea europaea* Leaves Extract and its Antibacterial Activity. Nanosci Nanotechnol. 2013;2:164–70.
